# A Biomimetic In Vitro Model of the Pro-Inflammatory Microenvironment Recapitulates Type I/II Interferon Signaling and Refractory Acute Monocytic Leukemia

**DOI:** 10.3390/biology15201876

**Published:** 2026-10-17

**Authors:** Kirill Krasnov, Margarita Kobyakova, Elena Meshcheriakova, Yana Lomovskaya, Yuliya Baburina, Olga Krestinina, Anatoly Senotov, Irina Fadeeva, Raif Vasilov, Roman Fadeev

**Affiliations:** 1 Institute of Theoretical and Experimental Biophysics, Russian Academy of Sciences, 142290 Pushchino, Russia; kobyakovami@gmail.com (M.K.); elena.mesh2311@gmail.com (E.M.); yannalomovskaya@gmail.com (Y.L.); byul@rambler.ru (Y.B.); ovkres@mail.ru (O.K.); a.s.senotov@gmail.com (A.S.); aurin.fad@gmail.com (I.F.); fadeevrs@gmail.com (R.F.); 2 National Research Center “Kurchatov Institute”, 123182 Moscow, Russia; raifvasilov@mail.ru

**Keywords:** acute monocytic leukemia (AMoL; AML M5), interferons, drug resistance, pro-inflammatory microenvironment, in vitro model

## Abstract

Preclinical drug development suffers from a persistent translational gap, as conventional animal models fail to reproduce the human immune response, contributing to high attrition rates of drug candidates in clinical trials. To address this, we previously established an in vitro high-density culture (HDC) model of acute monocytic leukemia (AML) cells that recapitulates an aseptic pro-inflammatory microenvironment using acute monocytic leukemia (AMoL) THP-1 cells. In the present study, using RNA-seq, quantitative RT-PCR, flow cytometry, Western blotting, and multiplex cytokine analysis, we characterized how this biomimetic microenvironment shapes Type I and II interferon signaling in THP-1 cells that acquire resistance to apoptosis under HDC conditions. The model revealed predominant activation of the Type II interferon pathway, marked by elevated interferon-gamma expression, STAT1 activation, and increased production of interferon-stimulated chemokines. Protein–protein interaction network analysis identified key interferon-induced hub genes and regulatory transcription factors driving this response. Notably, the interferon-stimulated gene profile generated by this in vitro platform, including the Interferon-Related DNA Damage Resistance Signature, closely resembled gene expression patterns observed in clinical samples from patients with refractory AMoL, supporting the relevance of the HDC biomimetic system as a research model of inflammatory adaptation in leukemia cells and associated resistance. These findings support further investigation of the HDC biomimetic system as a research model of inflammatory adaptation in leukemia cells and associated resistance.

## Simple Summary

Acute monocytic leukemia is an aggressive blood cancer in which leukemia cells can avoid cell death, leaving patients with few effective options. To study this resistance, which is mediated exclusively by leukemia cells, we used a simple laboratory model based on one type of human leukemia cell. Grown at high density, these cells mimic crowded, inflammation-related features of the disease environment. Without added external stimuli, the cells themselves produce inflammation-promoting signals and become resistant to apoptosis, the cell’s built-in self-destruction program, which improves survival under cell-killing treatments. We studied interferons, which are immune messenger molecules, and found strong interferon-related activity, together with changes in genes and in transcription factors, which are proteins that control gene activity. The model’s gene activity pattern resembled that seen in patients whose leukemia no longer responds to treatment. Because the system uses a single cell type, these inflammation-related changes and the associated treatment resistance can be studied separately from other cell types. We used this model to examine in detail the molecular mechanisms of resistance mediated by leukemia cells themselves, including the complex interferon-associated signaling that accompanies this resistant state.

## 1. Introduction

Acute myeloid leukemia (AML) accounts for more than 80% of all acute leukemias diagnosed annually, while the overall five-year survival rate remains at the 30% level [1,2]. Low survival is largely due to chemoresistance and the resistance of leukemic cells to molecular effectors of anti-tumor immunity [3,4,5].

AML is a heterogeneous hematological disease with several classifiable subtypes. One of the most aggressive is subtype M5, corresponding to acute monocytic (AMoL) according to the French-American-British (FAB) system based on morphological and cytochemical characteristics of leukemic blasts [6,7]. Modern classification of AML relies primarily on the cytogenetic and molecular-genetic characteristics of leukemic cells. In current editions of the World Health Organization (WHO) and International Consensus Classification (ICC) classifications, acute myeloid leukemia with monocytic differentiation (AMoL, FAB M5 subtype) is often associated with *KMT2A* gene rearrangements, specifically the t(9;11)(p21.3;q23.3) translocation leading to the formation of the hybrid oncogene *KMT2A–MLLT3*. Approximately 15–30% of AMoL cases occur in childhood [6,8,9]. AMoL with *KMT2A* rearrangements is associated with a very poor prognosis, high recurrence rates, and a significant risk of forming resistant clones [8,10,11]. Despite the advances in modern pharmacology and hematology, induction chemotherapy remains the cornerstone of AMoL treatment; however, even upon achieving remission, a relapse occurs in a significant proportion of patients, the key mechanism of which is the development of drug resistance in leukemic cells.

Bone marrow in AML is a damaged tissue with signs of chronic inflammation, which, by activating signaling pathways for tumor cell survival, can promote their evasion of induction of death and is associated with a poor prognosis of the disease [6,12,13].

Of particular interest in this context are interferons (IFN)—a family of pleiotropic cytokines that play an important role in regulating innate and adaptive immune responses. These cytokines are divided into three types: type I (IFN-α, IFN-β, IFN-ε, IFN-κ, IFN-ω), type II (IFN-γ), and type III (IFN-λ), which differ in the chromosomal location of their genes, producer cells, receptors, and biological effects [14]. Currently, type I (α/β) and type II (γ) IFNs play a special role in oncology research due to their dual nature [15,16,17]. In AML, on the one hand, interferons are capable of inducing cytotoxic and cytostatic effects against leukemic cells. On the other hand, prolonged interferon-mediated signaling, especially via IFN-γ, can enhance tumor cell survival and promote the formation of a local immunosuppressive microenvironment in pathologically altered bone marrow [18,19]. Meanwhile, the molecular mechanisms underlying this phenomenon of dual interferon influence on AML cells and bone marrow microenvironment components remain not fully elucidated. Therefore, identifying the signaling networks induced by α/β and γ interferons is crucial for a deeper understanding of the mechanisms regulating AML cell survival mediated by the pro-inflammatory microenvironment.

In previous studies, we demonstrated that AML cells in high-density cultures (HDC), where leukemic cells autonomously generate an aseptic, survival-promoting milieu through homotypic auto-/paracrine interactions without exogenous inflammatory stimulation, exhibit enhanced production of pro-inflammatory cytokines and chemokines, as well as increased resistance to apoptosis, mimicking drug resistance mediated by the pro-inflammatory microenvironment [20,21]. Additionally, it has been established that these cells are characterized by mitochondrial dysfunction, which can result in chronic activation of intracellular pattern recognition receptors and reinforcement of interferon-associated signaling pathways [22,23,24].

The present study focuses on comprehensive transcriptomic analysis of type I and type II interferon-associated signaling as a key mediator linking inflammation and mitochondrial dysfunction in a biomimetic in vitro model of the microenvironment associated with AMoL.

## 2. Materials and Methods

### 2.1. Protocol of TRAIL Preparation

To obtain a soluble trimeric form of the TRAIL protein, we synthesized the isoleucine zipper motif and the *TRAIL* gene and cloned them into the plasmid vector (Novagen, Madison, WI, USA). The resulting gene was used to transform *E. coli* BL21 (DE3) cells, and a trimeric form of TRAIL with a molecular weight of 80 kDa was obtained by microbial synthesis, followed by purification by metal affinity chromatography [25].

### 2.2. Cell Cultures

Human AML cell lines THP-1 (TIB-202), MV4-11 (CRL-959), and HL60 (CCL-240) were obtained from ATCC (Manassas, Virginia, USA). The cells were maintained in RPMI-1640 medium (Sigma-Aldrich, St. Louis, MO, USA) supplemented with 10% fetal bovine serum (FBS; Gibco, Thermo Fisher Scientific, Waltham, MA, USA) and 40 μg/mL gentamicin sulfate (Sigma-Aldrich, St. Louis, MO, USA) at 37 °C in a humidified atmosphere of 5% CO_2_. The cell cultures were tested for mycoplasma infection using the MycoFluor™ Mycoplasma Detection Kit (Thermo Fisher Scientific, Waltham, MA, USA), and no mycoplasma was detected. To obtain low-density cultures (LDC) of cells, 5 × 10^3^ cells were seeded in 100 μL of culture medium per well of a U-bottom 96-well plate (Corning Inc., Corning, NY, USA). After 24 h, the cell count increased to 1.0 ± 0.1 × 10^4^ cells per well (equivalent to 1.0 ± 0.1 × 10^5^ cells/mL). To generate three-dimensional high-density cultures simulating pro-inflammatory cell activation (HDC), 5 × 10^3^ cells were seeded in 100 μL of medium per well in a U-bottom 96-well plate and cultured for 5 days without medium change. At the end of the culture period, each well contained a single three-dimensional multicellular aggregate with a cell count of 1.0 ± 0.1 × 10^5^ cells (equivalent to 1.0 ± 0.1 × 10^6^ cells/mL) [20,21,26].

Cells stimulated with lipopolysaccharide (LPS), a known pro-inflammatory activator, were also included in the study [27,28,29]. For this purpose, cells were seeded at a density of 5 × 10^4^ cells/mL in a culture medium containing 1 µg/mL of LPS from *E. coli* O111:B4 (Santa Cruz Biotechnology, Santa Cruz, CA, USA) and incubated for 24 h. After 24 h, the cell density increased to 9.0 ± 0.5 × 10^4^ cells/mL.

### 2.3. Assessment of Cellular Viability

The cells were taken from cultures after growth for 3 days and seeded in U-shaped wells of a 96-well plate at a density of 5 × 10^3^ cells in 100 μL of growth medium per well. The recombinant protein TRAIL, cytarabine (Sigma-Aldrich, St. Louis, MO, USA) and doxorubicin hydrochloride (Sigma-Aldrich, St. Louis, MO, USA) were added to cultures 24 h (LDC, 1 × 10^4^ cells) or 120 h (HDC, 1 × 10^5^ cells) after cell seeding. The cytotoxicity was assessed by the ratio of the number of living cells in the experimental and control (non-treated) cultures at 24 h after drug addition. The number of viable cells was determined by measuring the fluorescence intensity of resorufin, the product of resazurin reduction. The cells were incubated with resazurin (30 µg/mL) for 4 h at 37 °C and 5% CO_2_, and then the fluorescence intensity of the incubation medium was measured at Ex.532 nm/Em.590 nm using an infinite F200 plate reader (Tecan, Männedorf, Switzerland) [20,21,30].

### 2.4. Analysis of Phosphatidylserine Externalization

To study the externalization of phosphatidylserine, the cells were harvested from culture plates and washed in cell-staining buffer (BioLegend, San Diego, CA, USA), 300 g, for 5 min. Staining was performed using a 1 μg/mL Annexin V FITC Conjugate (Lumiprobe, Moscow, Russia). The staining was carried out at room temperature in the dark for 20 min, and an analysis of Annexin V-positive cells was performed using a BD Accuri C6 flow cytometer (BD Bioscience, Franklin Lakes, NJ, USA). Histograms showing Annexin V-FITC expression were generated and analyzed using FlowJo software (version 10.8.1; FlowJo LLC, Ashland, OR, USA).

### 2.5. RNA Sequencing

Bulk RNA transcriptome sequencing was conducted at «Genoanalytika» LLC utilizing the HiSeq1500 Illumina next-generation sequencer (Illumina, San Diego, CA, USA).

Total RNA was extracted from cell lysates using Trisol reagent (Thermo Fisher Scientific, Waltham, MA, USA) and the PureLink RNA Micro Kit (Invitrogen, Waltham, MA, USA). RNA quality/quantity assessment was performed via BioAnalyzer and the RNA 6000 Nano Kit (Agilent Technologies, Santa Clara, CA, USA). PolyA RNA isolation was performed using Dynabeads^®^ Oligo(dT) magnetic beads (Invitrogen, Waltham, MA, USA).

Library preparation with NEBNext^®^ Ultra™ RNA Library Prep Kit for Illumina^®^ (New England Biolabs GmbH, Frankfurt, Germany). Library quantification using Qubit dsDNA HS Assay Kit (Thermo Fisher Scientific, Waltham, MA, USA). Fragment length distribution analysis with Agilent High Sensitivity DNA Kit (Agilent Technologies, Santa Clara, CA, USA). Sequencing on HiSeq1500 with ≥10 million 50-nucleotide reads. Read alignment to the reference genome and quantification were performed using STAR (v.2.5.3a).

We used 3 biological replicates for each condition to produce RNA sequencing analysis. For all RNA-seq data used, quality control was performed using FastQC (v. 0.12.1) and MultiQC (v. 1.17) software (Table S1, Figure S1A–D).

Data can be obtained from the Gene Expression Omnibus database using the identifier GSE298747.

### 2.6. Analysis of Differential Gene Expression

A differential gene expression analysis was conducted utilizing DESeq2 (v. 1.38.3) within the RStudio (v. 2024.04.0+735) R environment (v. 4.4.2) [31]. The study examined the differential gene expression between THP-1 AMoL cells cultured at high density, which are resistant to apoptosis inducers, and those cultured at low density, which are sensitive to apoptosis inducers. In our study, we compared untreated high-density cell (HDC) cultures with low-density cell (LDC) cultures for a detailed analysis of gene expression differences. We also conducted principal component analysis (PCA) and calculated the Euclidean distance matrix between samples (Figure S1E,F). For both PCA and the sample-to-sample pairwise distance matrix, we utilized all differentially expressed genes to assess the relationships among samples through multivariate decomposition of variance.

The RNA sequencing data from patients diagnosed with AMoL (according to the FAB classification) were obtained from the open database of the beatAML project [32]. For this study, we selected results from the RNA sequencing of 55 samples: 14 samples came from patients with refractory AMoL (also referred to as treatment-resistant), and 41 samples were from patients who demonstrated a strong response to therapy (referred to as treatment-sensitive). These selected samples are notable for harboring the t(9;11)(p22;q23) translocation, which leads to the formation of the chimeric *MLLT3-KMT2A* gene, and they also include an *NRAS* p.G12D mutation.

### 2.7. Gene Set Enrichment Analysis (GSEA)

We conducted Gene Set Enrichment Analysis utilizing gene sets from the Transcription Factor Targets (TFTs), Hallmark, Reactome, Gene Ontology: Biological Processes (GO:BP), BioCarta, and WikiPathways (WP) collections acquired from the Molecular Signatures Database (MSigDB, v. 2023.1, https://www.gsea-msigdb.org/gsea/index.jsp, accessed on 26 June 2024). The GSEApy (v. 1.1.6) package in Python (v. 3.11.7) was utilized for this purpose [33,34].

The Interferon-Related DNA Damage Resistance Signature (IRDS) gene set was collected from published articles [35,36,37] (Table S2) with the following GSEA.

### 2.8. Analysis of Interferon-Stimulated Genes

The expression of ISGs was evaluated utilizing data from the Interferome database (v. 2.1), which was acquired through the ISGPRE service (https://isgpre.cvr.gla.ac.uk/, accessed on 21 October 2025) [38]. This enabled us to identify genes that induce IFN I and II types, exhibiting statistically significant alterations in gene expression levels.

### 2.9. Networks of Protein–Protein Interaction

To visualize and analyze the complex interactions among genes implicated in AMoL, we constructed protein–protein interaction (PPI) networks using stable Ensembl IDs from the STRING database (v. 12.0, https://string-db.org/, accessed on 16 March 2026). These networks were visualized with Cytoscape software (v. 3.10.3, https://cytoscape.org, accessed on 26 November 2024) [39,40]. Our analysis focused on interconnected gene components, excluding individual nodes that represented specific genes to emphasize the relationships among them. We applied network clustering using the cytoHubba module (v. 0.1) within the Cytoscape environment [41]. We employed both global and local clustering methods to identify key genes or «hub-genes» which play crucial roles in the observed interactions.

### 2.10. Functional Enrichment Assessment

We employed the Reactome database, available at https://reactome.org/ (accessed on 17 March 2026), for the functional enrichment analysis of identified interferon-stimulated genes [42]. The Enrichr service was utilized to do a more in-depth analysis of these gene sets via its API (https://maayanlab.cloud/Enrichr/, accessed on 17 March 2026) [43].

### 2.11. Analysis of Transcription Factors

To identify potential transcription factors that might modulate the expression of the key genes or «hub-genes» identified in our study, we utilized data from the C3 collection within the MSigDB database, specifically focusing on the Transcription Factor Targets (TFTs) subset (https://www.gsea-msigdb.org, accessed on 26 June 2024). We screened for a minimum of one target gene among these hubs to pinpoint potential transcription factors. To streamline our analysis and avoid redundancy, we consolidated related sets while eliminating any duplicates.

### 2.12. Cytokines Assay

Culture medium was taken and centrifuged (300× *g*, 5 min) one day after cell seeding at a concentration of 5 × 10^4^ cells/mL or 5 days after cell seeding in U-shaped wells of a 96-well plate. The content of IFNγ, IP-10, MCP-1, MIP-1α, MIP-1β, and RANTES in the supernatant was evaluated with a Bio-Plex Pro Human Cytokine Grp I Panel 27-plex kit (Bio-Rad, Hercules, CA, USA) using a Bio-Plex MAGPIX multiplex analyzer (Bio-Rad, Hercules, CA, USA). Cytokines/chemokines concentration normalized to 1 × 10^4^ cells.

### 2.13. Flow Cytometry Analysis of Interferon Receptors (IFNRs)

For analysis, the cells were harvested from culture flasks and washed in cell-staining buffer (BioLegend, San Diego, CA, USA) at 300× *g* for 5 min. Staining was performed using a panel of monoclonal antibodies: IFNAR1 PE (FHD23012), CD119/IFNGR1 FITC (FHD09811), and IFNGR2 APC (FHE22713) (AntibodySystem, Paris, France) and IFNAR2 FITC (10359-R122-F; Sino Biological, Beijing, China). To assess the possibility of non-specific antibody binding, cells were stained with antibodies IgG2a kappa APC, IgG1 kappa PE (BioLegend, San Diego, CA, USA), and IgG1 kappa FITC (eBioscience, San Diego, CA, USA), respectively. Staining was carried out at room temperature in the dark for 30 min. After staining, the cells were fixed with a 2% paraformaldehyde solution, and an analysis was performed using a BD Accuri C6 flow cytometer (BD Bioscience, San Jose, CA, USA).

### 2.14. Western Blot Analysis

To obtain cell lysate, cells were washed with cold phosphate-buffered saline (300× *g*, 5 min), and the cell pellet was dissolved in RIPA buffer (Santa Cruz Biotechnology, Santa Cruz, CA, USA) containing protease inhibitor cocktail (Santa Cruz Biotechnology, Santa Cruz, CA, USA) and phenylmethylsulfonyl fluoride (PMSF, Servicebio, Wuhan, Hubei, China) for 305 min on ice. Samples were centrifuged at 13,000× *g* for 10 min. Protein concentration was determined in the supernatant using the Bradford method with Pierce Detergent Compatible Bradford Assay Reagent (Thermo Fisher Scientific, Waltham, MA, USA) according to the manufacturer’s instructions [44]. Bovine serum albumin (BSA) standards were prepared in the same RIPA buffer as the samples to minimize matrix effects.

Electrophoretic separation of proteins was performed in 10%, 12.5%, or 15% polyacrylamide gels, depending on the size of the protein under analysis, using a Mini-Protan Hoefer chamber (Pharmacia Biotech, Uppsala, Sweden) and a PowerPac Universal Power Supply (Bio-Rad, Hercules, CA, USA). Protein transfer from the gel to 0.2 μm nitrocellulose membranes (Bio-Rad, Hercules, CA, USA) was performed using the Trans-Blot Turbo system with the Trans-Blot Turbo Transfer Kit (Bio-Rad, Hercules, CA, USA) according to the manufacturer’s instructions. Blocking of non-specific binding on membranes was performed in Roti-Block solution (Carl Roth GmbH + Co., Karlsruhe, Germany). Membranes were blocked with Roti-Block solution (Carl Roth GmbH + Co., Karlsruhe, Germany) or 5% bovine serum albumin (BSA) in Tris-buffered saline with 0.1% Tween 20 (TBST) (VWR International, Radnor, PA, USA) at room temperature for one hour. Staining with primary antibodies was carried out overnight at +4 °C. The following primary antibodies were used: IRF9 (#76684), STAT1 (#14994), pSTAT1 (#7649, Tyr701), STAT2 (#72604), and pSTAT2 (#88410, Tyr690, Cell Signaling, Danvers, MA, USA) at a dilution of 1:1000 according to the manufacturer’s instructions. GAPDH (sc-365062, Santa Cruz Biotechnology, Santa Cruz, CA, USA) was used as a loading control at a dilution of 1:2000.

Subsequently, incubation with corresponding secondary antibodies conjugated to horseradish peroxidase (HRP) (Jackson ImmunoResearch, West Grove, PA, USA) was performed for one hour at room temperature. Goat Anti-Rabbit IgG (Bio-Rad, Hercules, CA, USA) and Anti-Mouse IgG (Cell Signaling, Danvers, MA, USA) were used as secondary antibodies at dilutions of 1:20,000 and 1:5000, respectively. Chemiluminescent detection of proteins was performed using the ChemiDoc MP imaging system (Bio-Rad, Hercules, CA, USA) with Clarity Western ECL substrate (Bio-Rad, Hercules, CA, USA). Quantitative analysis of protein band intensities was performed by densitometry using Image Lab 6.0 software (Bio-Rad, Hercules, CA, USA).

Original membranes with Western blot results are shown in Figure S2.

### 2.15. DNA Copy Number Variation (CNV) Analysis

DNA copy number profiling was performed using Agilent Sure Print G3 Human CGH microarray 180 K (Agilent Technologies, Santa Clara, CA, USA). All experiments were conducted according to the manufacturer’s instructions. In brief, 1 µg of genomic DNA from THP-1 cells was labeled with Cy5-dCTP (Sigma-Aldrich, Hamburg, Germany) and reference human DNA was labeled with Cy3-dCTP (Sigma Aldrich, Hamburg, Germany). Labeled DNA was applied to the array with hybridization buffer and human Cot-1 DNA (Thermo Scientific, Waltham, MA, USA). Array slides were incubated for 24 h at 65 °C. After washing and scanning the arrays, images were analyzed with Feature Extraction software (v. 10.7.3.1, Agilent Technologies, Santa Clara, CA, USA). Probe mapping was conducted according to its genomic location in the UCSC genome browser (human NCBI37/hg19). The Rank Segmentation statistical algorithm in NEXUS software (v. 7.5, Biodiscovery Inc., El Segundo, CA, USA) was used to define copy number alterations in each sample. Results are presented in Table S3.

### 2.16. Quantitative Real-Time PCR (qRT-PCR)

DNA oligonucleotide primers were designed using Primer-BLAST software (https://www.ncbi.nlm.nih.gov/tools/primer-blast/, accessed on 14 October 2026) (National Library of Medicine, Bethesda, MD, USA) considering the presence of introns and potential alternative splicing variants, and synthesized by JSC Evrogen; they are presented in Table 1. Total RNA extraction was performed using the SKYEasy RNA Fast Tissue/Cell Kit (SkyGen, Guangzhou, China). cDNA synthesis and amplification were performed using the OneTube RT-PCR SYBR kit (Evrogen, Moscow, Russia) on a QuantStudio 5 Real-Time PCR instrument (Thermo Fisher Scientific, Waltham, MA, USA) with QuantStudio™ Design & Analysis Software (v. 1.5.2, Thermo Fisher Scientific, Waltham, MA, USA). Expression levels were quantified by measuring cycle threshold (Ct) values and normalized to GAPDH. To measure changes in gene expression, the RT2 Profiler PCR Data Analysis web service (Quagen, Venlo, Netherlands) was used, and fold change in each sample compared to the control group was calculated.

### 2.17. Statistical Analysis

Results are expressed as mean ± standard deviation (M ± SD), with all tests conducted in at least three replicates (*n* ≥ 3).

For proteins measured using ELISA, Western blot and flow cytometry data, statistical analysis was performed independently for each analyzed variable to assess differences between experimental sample groups. Comparisons were made between samples within the same group using Welch’s ANOVA with Dunnett’s (p.adj) test and global Benjamini–Hochberg false discovery rate (FDR) correction. The qRT-PCR results were processed using the Kruskal–Wallis method with Dunn’s correction (p.adj) due to the pronounced asymmetry of variance.

We used the Wald test to evaluate differential gene expression, incorporating Benjamini–Hochberg false discovery rate (FDR) correction. Survival analysis for significance of differences, conducted using the GEPIA service, is presented as the log-rank test. Transcriptomic results are reported only when meeting a significance threshold of FDR ≤ 0.05.

SciPy (v. 1.11.4), NumPy (v. 1.26.2), Pandas (v. 2.1.3), and statsmodels (v. 0.14.0) libraries were used for statistical testing [44,45,46,47]. Matplotlib (v. 3.10.0), seaborn (v. 0.13.1) and pyCirclize (v. 1.9.0) libraries were used for data visualization [48,49].

## 3. Results

### 3.1. Analysis of Interferon-Induced Signaling in Apoptosis-Resistant AMoL THP-1 Cells in In Vitro Model of a Pro-Inflammatory Microenvironment

**Figure 1:**
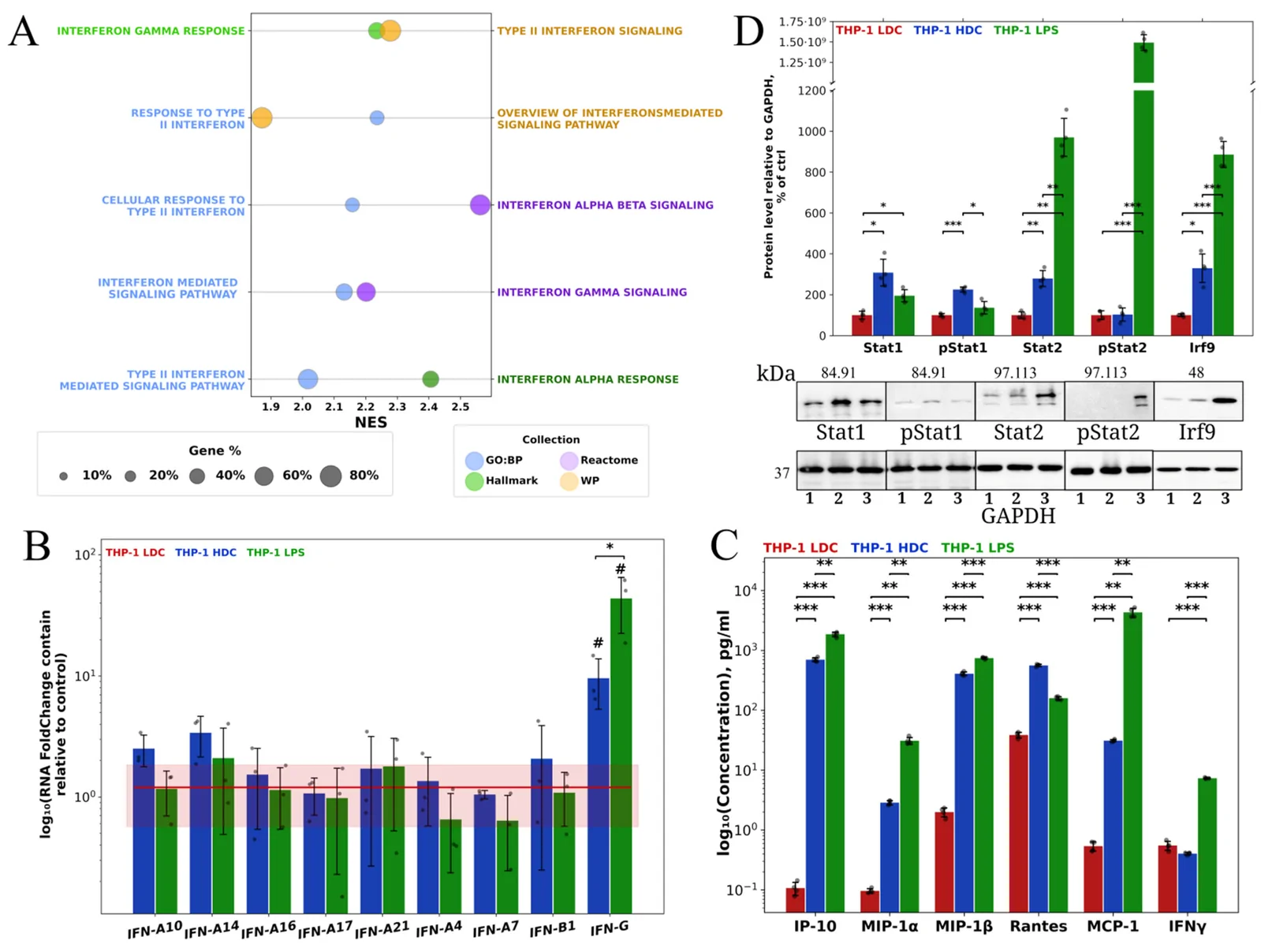
Activation of interferon-induced signaling and JAK/STAT signaling pathway in apoptosis-resistant THP-1 cells under a pro-inflammatory microenvironment. (**A**) GSEA of gene sets associated with type I and II interferon signaling pathways from the Reactome, Gene Ontology: Biological Process (GO:BP), Hallmark, and WikiPathways (WP) collections in THP-1 HDC cells compared to THP-1 LDC cells (color indicates the collection; shade represents the axis on which the gene set is located). (**B**) Content of mRNA of various *IFN-alpha*, *IFN-B1*, and *IFN-G* isoforms in THP-1 LDC, THP-1 HDC, and THP-1 LPS cell cultures (*—p.adj ≤ 0.05; #—p.adj ≤ 0.05 according to THP-1 LDC). The red line shown throughout the plot represents the mean, and the red shaded area represents the ± SD of the control samples. (**C**) Content of interferon-induced chemokines) and type II (IFN-gamma) interferon in THP-1 LDC, THP-1 HDC and THP-1 LPS cell culture. (**D**) Western blot analysis results of components of the STAT signaling pathway in THP-1 LDC (1), THP-1 HDC (2) & THP-1 LPS (3) cells. GSEA—FDR ≤ 0.05; *—FDR ≤ 0.05; **—≤ 0.01; ***—≤ 0.001.

To analyze interferon-induced signaling pathways, we used AMoL THP-1 cells resistant to apoptosis induction when cultured in high-density culture (THP-1 HDC), mimicking a pro-inflammatory microenvironment, and compared them with the same cells cultured under low-density conditions and sensitive to apoptosis induction (THP-1 LDC) (Figure S3A) [20]. A similar effect was also demonstrated for AML MV4-11 and HL-60 cells (Figure S3B,C) [20]. To assess the activation of interferon-associated signaling in THP-1 HDC, a Gene Set Enrichment Analysis (GSEA) was performed using interferon signaling pathway gene sets from the MSigDB database. The analysis included the «Interferon Alpha Beta Signaling» and «Interferon Gamma Signaling» gene sets from the Reactome collection; «Response to Type II Interferon», «Cellular Response to Type II Interferon», «Interferon Mediated Signaling Pathway», and «Type II Interferon Mediated Signaling Pathway» from the Gene Onthology: Biological Processes (GO:BP) collection; «Type II Interferon Signaling» and «Overview of Interferons Mediated Signaling Pathway» from the WikiPathways (WP) collection; as well as «Interferon Alpha Response» and «Interferon Gamma Response» from the Hallmark collection. GSEA showed that all specified gene sets had a positive normalized enrichment score (NES) in THP-1 HDC cells compared to THP-1 LDC cells, indicating the activation of corresponding interferon-induced signaling pathways in apoptosis-resistant THP-1 HDC cells (Figure 1A).

Since enrichment of interferon-induced gene sets was identified in apoptosis-resistant AMoL cells, we further evaluated the expression of type I (alpha and beta) and type II (gamma) IFN genes using qRT-PCR. As a control for the inflammatory response, THP-1 cells treated with lipopolysaccharide (THP-1 LPS) were used, for which increased resistance to apoptosis induction has been previously shown [20,21,30]. Data exist that AMoL THP-1 cells are characterized by a homozygous deletion of 9p21 and, consequently, the absence of regions containing *IFNA1*, *IFNA2*, *IFNA5*, *IFNA6*, *IFNA8*, and *IFNA13* genes [50]. To confirm these data, comparative genomic hybridization was performed to identify copy number variation (CNV) in the AMoL THP-1 cells used in this study. The results are presented in Table S3. The obtained results confirm that the THP-1 cells used in this study possess the same homozygous deletion described in the aforementioned work. In this regard, only the expression of *IFNA4*, *IFNA7*, *IFNA10*, *IFNA14*, *IFNA16*, *IFNA17*, and *IFNA21*, as well as *IFNB1* and *IFNG*, was studied. It was shown that the expression of *IFNA4*, *IFNA7*, *IFNA10*, *IFNA14*, *IFNA16*, *IFNA17*, *IFNA21*, and *IFNB1* in apoptosis-resistant THP-1 HDC cells did not change compared to apoptosis-sensitive THP-1 LDC cells. At the same time, the expression of the *IFNG* gene in apoptosis-resistant THP-1 HDC cells was increased compared to apoptosis-sensitive THP-1 LDC cells (*p* ≤ 0.05) (Figure 1B). Similar results were obtained for LPS-treated cells (Figure 1B). At the same time, we observed an increase in extracellular levels of interferon-induced chemokines such as monocyte chemoattractant protein 1 (MCP-1; CCL2), macrophage inflammatory proteins MIP-α and -β (CCL3/4), regulated on activation, normal T-cell expressed and secreted (Rantes; CCL5), and interferon gamma-induced protein 10 (IP-10; CXCL10) compared to apoptosis-sensitive THP-1 LDC cells. Furthermore, we observed higher extracellular IFNγ levels in THP-1 LPS cells than in THP-1 HDC cells, compared with THP-1 LDC cells (Figure 1C, Table S4), which may indicate a lower secretion level in THP-1 HDC cells compared to THP-1 LPS and/or faster redistribution by cells.

The next step was to evaluate the surface expression of interferon receptors IFNAR1, IFNAR2, IFNGR1, and IFNGR2 in apoptosis-resistant THP-1 HDC cells compared with apoptosis-sensitive THP-1 LDC cells and LPS-treated THP-1 cells. A decrease in the surface expression of IFNAR1, but not IFNAR2, IFNGR1, and IFNGR2, was shown in THP-1 HDC and THP-1 LPS cells compared to THP-1 LDC cells (Figure S4A).

It is known that the key intracellular signal transducers of interferons are STAT transcription factors [15,51]. Next, using GSEA, an assessment of transcriptional activation of genes in the JAK/STAT signaling pathway was performed in apoptosis-resistant THP-1 HDC cells. Positive enrichment of gene sets «Positive regulation of receptor signaling pathway via STAT», «Receptor signaling pathway via STAT», «Regulation of receptor signaling pathway via STAT», and «JAK/STAT Signaling pathway» from the GO:BP and KEGG collections was identified, indicating activation of these signaling pathways (Figure S4B).

Furthermore, an assessment of key proteins in the interferon-induced STAT signaling pathway was performed in apoptosis-resistant THP-1 HDC cells. Figure 1D shows that in apoptosis-resistant THP-1 HDC cells compared to apoptosis-sensitive THP-1 LDC cells, there was an increase in the levels of signal transducer and activator of transcription 1 (Stat1) and its phosphorylated form pStat1 (Tyr701). On the other hand, in apoptosis-resistant THP-1 HDC cells, there was an increase in the level of signal transducer and activator of transcription 2 (Stat2), with a complete absence of the phosphorylated form pStat2 (Tyr690). Additionally, a significant increase in the interferon regulatory factor 9 (Irf9) was identified in apoptosis-resistant THP-1 HDC cells. In turn, in THP-1 LPS cells, an increase in Stat1 and Stat2, as well as their phosphorylated forms pStat2 (Tyr690), but not pStat1 (Tyr701), was observed, along with an increase in the Irf9 protein compared to THP-1 LDC cells (Figure 1D). The Western blotting results indicate that in apoptosis-resistant THP-1 HDC cells, activation of the interferon gamma-induced signaling pathway leading to the formation of a STAT1 homodimer known as GAF (Gamma-Activated Factor) is possible [52], while in THP-1 LPS cells, activation of interferon I and II signaling pathways based on both GAF formation and the classical trimeric complex STAT1–STAT2–IRF9 known as Interferon-Stimulated Gene Factor 3 (ISGF3) may occur [53,54,55].

Thus, for apoptosis-resistant THP-1 cells in an in vitro model of a pro-inflammatory microenvironment, it is characteristic to have positive enrichment of interferon-induced gene sets, increased expression of the *IFNG* gene, phosphorylation of the transcription factor STAT1, and accumulation of extracellular forms of interferon-induced chemokines, which may be associated with the activation of interferon-associated signaling pathways, most likely mediated by interferon gamma.

### 3.2. Analysis of Differential Expression of Interferon-Stimulated Genes (ISGs) in Apoptosis-Resistant AMoL THP-1 Cells in a Pro-Inflammatory Microenvironment

**Figure 2:**
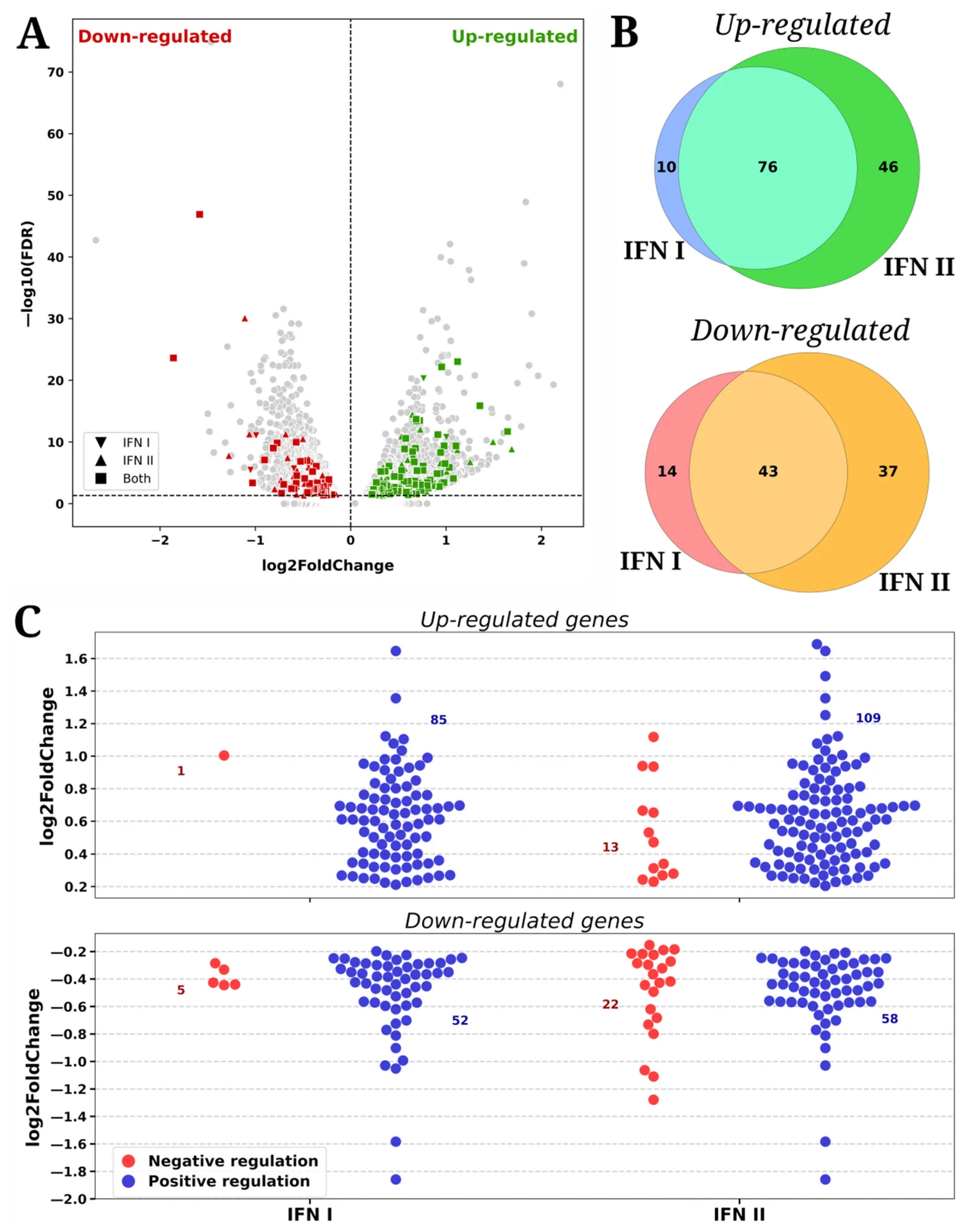
The examination of differential gene expression (DGE) induced by type I and II interferons in THP-1 HDC cells. (**A**) Distribution of ISGs based on their log2FoldChange and the significance of their expression changes (−log10(FDR)). (**B**) Histograms illustrating the distribution of genes activated by type I Interferons (**left side**) and type II interferons (**right side**), with increased (**top**) and decreased (**bottom**) expression in THP-1 HDC cells. (**C**) DGE elicited by type I interferons (**left**) and type II interferons (**right**) in THP-1 HDC cells, in accordance with «canonical» gene expression regulation. |log2FoldChange| > 0; FDR ≤ 0.05.

Next, a detailed analysis of the differential expression of interferon-stimulated genes (ISGs) in apoptosis-resistant THP-1 HDC cells compared to apoptosis-sensitive THP-1 LDC cells was performed using the Interferome database. It was found that 226 ISGs induced by type I and II interferons exhibited differential expression in THP-1 HDC cells compared to THP-1 LDC cells; among them, 132 genes were characterized by increased expression and 94 by decreased expression (Figure 2A). Furthermore, most of the identified genes are stimulated by both types of interferons: 76 genes with increased expression and 43 genes with decreased expression. Additionally, it was shown that a significant portion of these genes is specifically regulated by type II interferons. In total, 83 genes are ISGs induced by IFN II; of these, 46 show increased expression and 37 show decreased expression. In contrast, only 24 genes are activated exclusively by type I interferons, of which 10 have increased expression, and 14 have decreased expression (Figure 2B).

Analysis of the «canonical» regulation of gene expression induced by type I interferons according to the Interferome database showed that among 137 genes for which increased expression in response to IFN I is described, approximately two-thirds (85 genes) actually show increased expression in THP-1 HDC, while 52 show decreased expression. Among the group of six genes characterized by decreased expression upon stimulation with type I interferons, most (five genes) also showed decreased expression in THP-1 HDC, and only one gene showed increased expression compared to THP-1 LDC. In turn, when analyzing the «canonical» regulation of genes induced by type II interferons, it was found that among 167 genes whose expression increases upon exposure to IFN II, genes with increased expression predominate in THP-1 HDC (109 genes) compared to genes with decreased expression (58 genes). In the cohort of 35 genes for which decreased expression upon exposure to IFN II is described in the Interferome database, most (22 genes) also show decreased expression in THP-1 HDC, while 13 genes have increased expression (Figure 2C).

Overall, the identified ISGs expression patterns are consistent with canonical interferon-associated regulation described in the Interferome database and indicate a predominance of transcriptional changes associated with type II interferons. The predominant increase in the expression of genes induced by IFN II, along with a significant number of genes stimulated by both types of interferons, indicates the formation of a stable IFN II-associated transcriptional signature, which may contribute to maintaining the resistant phenotype under pro-inflammatory microenvironment conditions in leukemia cells.

### 3.3. Analysis of Protein–Protein Interaction Networks of Interferon-Stimulated Genes in Apoptosis-Resistant AMoL THP-1 Cells in a Pro-Inflammatory Microenvironment

To identify key (hub) genes, i.e., genes occupying a central position in signaling networks and having high connectivity with other genes, an analysis of protein–protein interaction (PPI) networks of the products of identified ISGs was performed. For this purpose, all identified ISGs were selected and divided into two groups: genes with increased expression (Figure S5A) and decreased expression (Figure S5B). Among the obtained PPIs, only genes forming a single interaction network were selected. As a result, for apoptosis-resistant THP-1 HDC cells compared to apoptosis-sensitive THP-1 LDC cells, 72 ISGs with increased expression and 43 ISGs with decreased expression were obtained.

**Figure 3:**
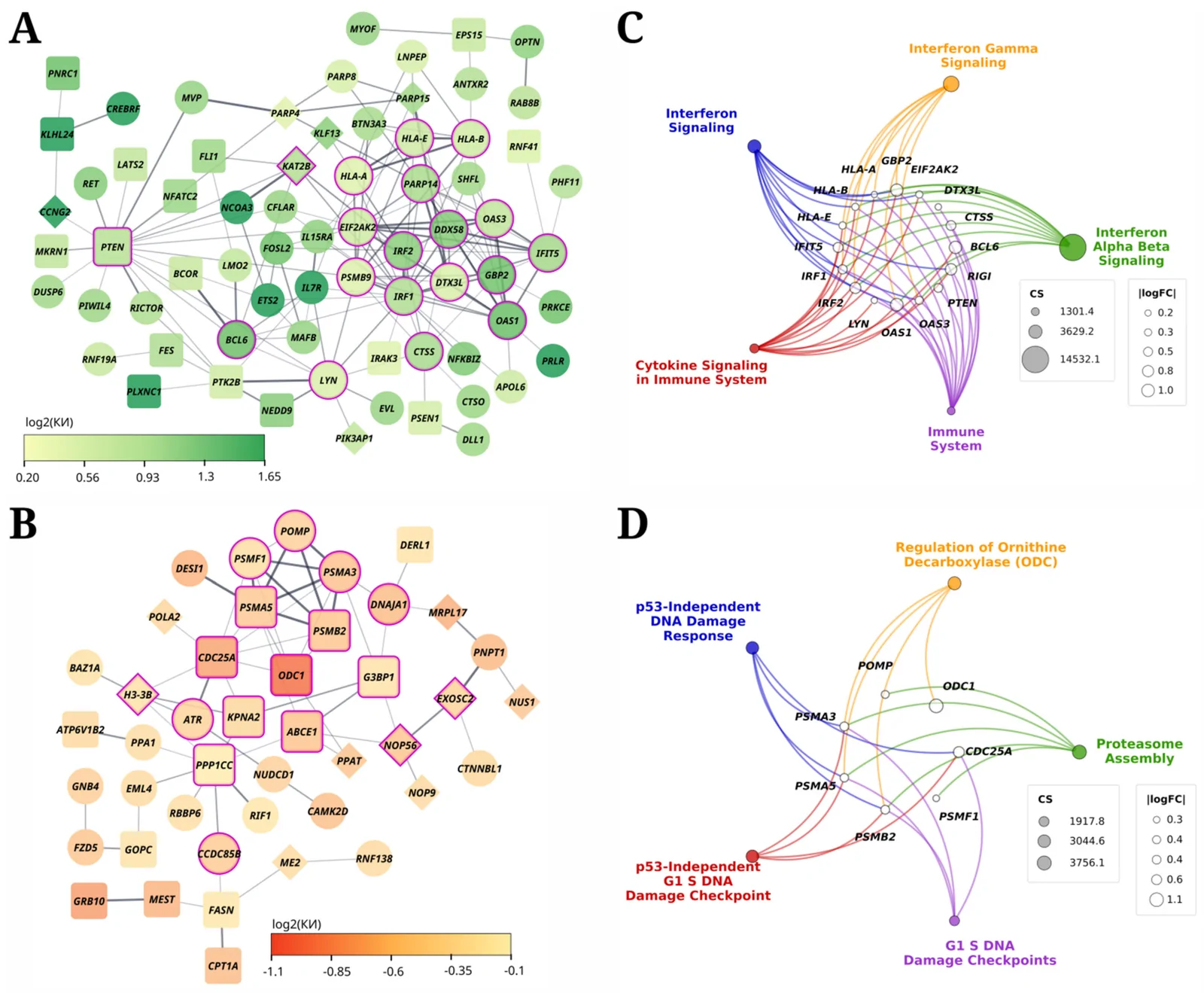
Protein–protein interaction networks among interferon-stimulated genes in THP-1 HDC cells. Protein–protein interaction (PPI) of interferon-stimulated genes exhibiting increased (**A**) and decreased (**B**) expression levels. Hub genes are highlighted by frames and sizes. The functional annotation of hub genes with increased (**C**) and decreased (**D**) expression is presented. Pathways: Reactome, FDR ≤ 0.05.

Clustering of the interaction networks for genes with increased expression revealed 18 hub genes associated with resistance to tumor cell death and suppression of the anti-tumor immune response. These genes included: oligoadenylate synthetases 1 and 3 (*OAS1*, *OAS3*) [56,57], interferon regulatory factors (*IRF1*, *IRF2*) [58,59], B-cell lymphoma protein (*BCL6*), interferon-induced protein with tetratripeptide repeats 5 (*IFIT5*) [60], cathepsin S (*CTSS*) [61], tyrosine kinase Lyn (*LYN*) [62], serine/threonine protein kinase (*EIF2AK2*) [63], ADP-ribose polymerase family 14 (*PARP14*) [64], RIG-I-like receptor (*DDX58*) [65], 20S proteasome subunit beta 9 (*PSMB9*) [66] and lysine acetyltransferase 2B (*KAT2B*) [67]. Furthermore, we identified genes that directly regulate or indirectly affect the expression levels of major histocompatibility complex (*MHC*) class I (*HLA-A*, *HLA-B*, *HLA-E*) [68], as well as phosphatase and tensin homolog (*PTEN*) [69] and guanylate-binding protein 2 (*GBP2*) [70] (Figure 3A).

In contrast, when clustering the networks of products of genes with decreased expression, we identified 17 hub genes whose products encode components of the proteasome complex, including constitutive 20S proteasome complex subunits (*PSMA3*, *PSMA5*, *PSMB2*) [71], proteasome inhibitor subunit PI31 (*PSMF1*) [72], proteasome maturation protein (*POMP*) [73] and an ABC transporter family ATPase (*ABCE1*) [74]. We also identified associations with serine/threonine protein phosphatase PP1-gamma (*PPP1CC*) [75], mutant ataxia-telangiectasia and Rad3-related protein (*ATR*) [76], histone H3 family 3B (*H3-3B*) [77], Ras GTPase-binding stress granule protein (*G3BP1*) [78], ribonucleoprotein 56 (*NOP56*) [79], M-phase phosphatase (CDC25A) [80], delta-interacting protein A (*CCDC85B*) [81], heat-shock protein 40 family co-chaperone (*DNAJA1*) [82], exosome component 2 (*EXOSC2*) [83], ornithine decarboxylase 1 (*ODC1*) [84] and karyopherin alpha 2 (*KPNA2*) [85] (Figure 3B).

After identifying the hub genes in the interaction networks of ISG products, we proceeded to their functional annotation. For this purpose, the Reactome database and the Enrichr web platform were used. From the 238 identified signaling pathways (42 for hub genes with increased expression and 196 for hub genes with decreased expression), five annotations with the highest combined score (activity coefficient × −log10(*p*-value)) were selected. Among the resulting sets of genes, for hub genes with increased expression, immune system processes mediated by type I and II interferons were identified (Figure 3C). For hub genes with decreased expression, functional associations were found for seven gene products involved in the regulation of p53-independent DNA damage response, assembly of proteasome complexes, and regulation of ornithine decarboxylase—a key enzyme in polyamine metabolism (Figure 3D), which is consistent with the classical understanding of interferon-associated signaling regulation [73,86,87,88,89].

Thus, the results presented indicate that for apoptosis-resistant THP-1 HDC cells in a pro-inflammatory microenvironment, a redistribution of interferon-induced networks toward enhanced hubs regulating interferon I/II-dependent immune and stress response signaling activity is characteristic, against a background of reduced activity of nodes responsible for proteasomal degradation, DNA damage control, and polyamine metabolism.

### 3.4. Identification of Transcription Factors Regulating the Expression of Hub ISGs in Apoptosis-Resistant AMoL THP-1 Cells in a Pro-Inflammatory Microenvironment

The next step was to identify transcription factors (TFs) associated with the identified interferon-stimulated genes and assess their transcriptional activity in apoptosis-resistant THP-1 HDC cells compared to apoptosis-sensitive THP-1 LDC cells.

**Figure 4:**
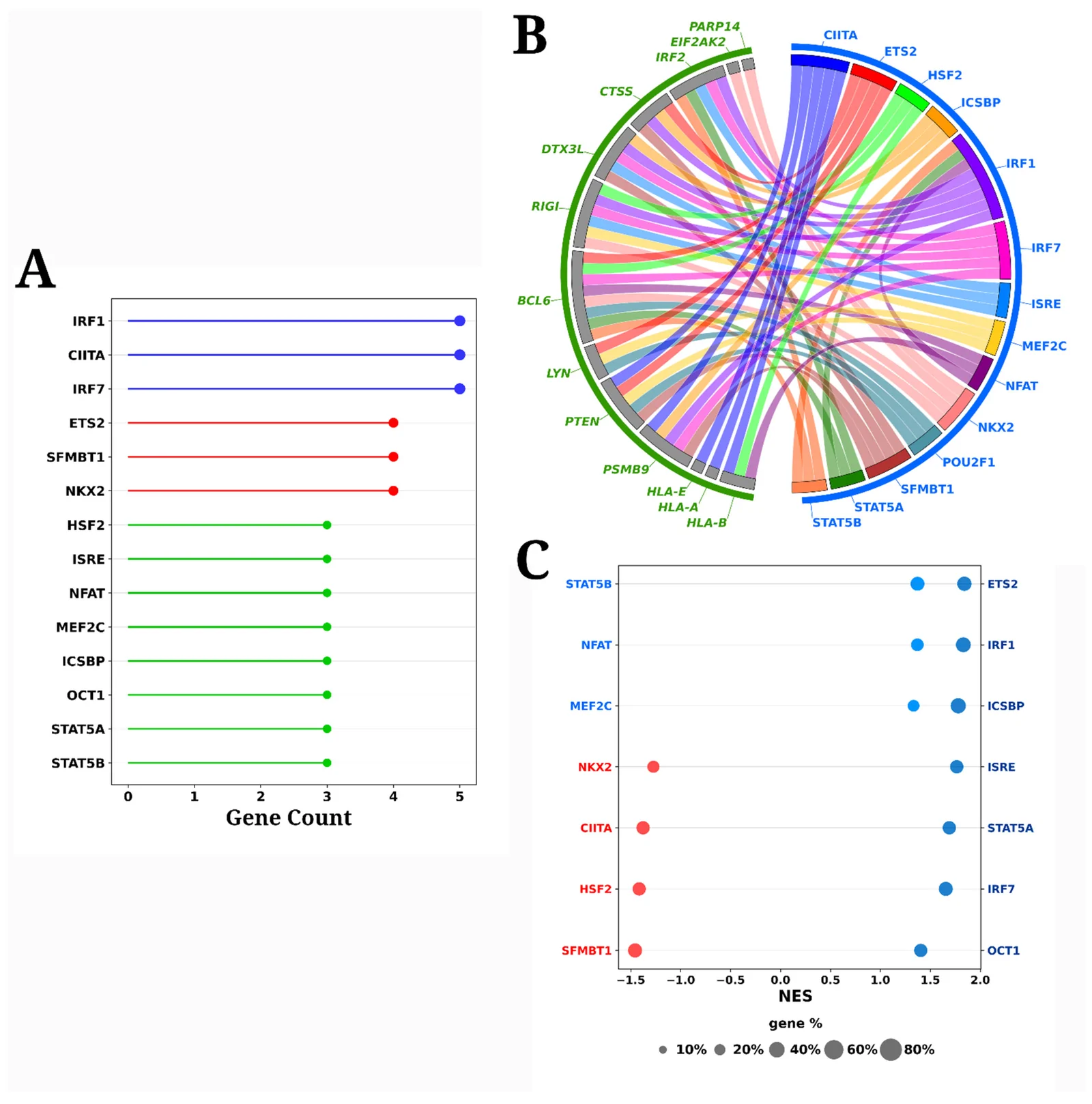
Examination of transcription factor (TF) activity associated with hub genes with increased expression in THP-1 HDC cells. (**A**) The quantity of hub genes regulated by transcription factors. (**B**) Association between discovered TFs (blue) and hub genes (green). (**C**) Gene Set Enrichment Analysis with identified transcription factors (colored dots represent gene collections; shading represents the axis on which each set is displayed). FDR ≤ 0.05.

A total of 44 TFs were identified, of which 14 were associated with genes having increased expression and 30 with genes having decreased expression. The identified TFs associated with ISGs showing increased expression included IRF1, CIITA, IRF7, ETS2, SFMBT1, NKX2, HSF2, ISRE (a complex consisting of STAT1, STAT2, and IRF9), and ICSBP (IRF8), with each being associated with at least three ISGs showing increased expression (Figure 4A). Chord diagrams reflecting the link between genes and TFs were constructed for the identified TFs and ISGs (Figure 4B). Next, a GSEA was performed for the identified TFs using gene sets from the TFT collection of the MSigDB database, showing positive enrichment for 10 transcription factor sets, which may indicate an increase in their activity. However, four transcription factor sets (NKX2, CIITA, HSF2, and SFMBT1) showed negative enrichment, which may indicate a decrease in their transcriptional activity (Figure 4C). It is known that the identified TFs, including STAT5A/5B, ISRE (a complex of STAT1, STAT2, and IRF9), IRF1/7, ICSBP (IRF8), and CIITA, can modulate pro-inflammatory interferon-dependent signaling pathways and the antiviral response [90,91,92,93]. In contrast, transcription factors NFAT, ETS2, MEF2C, and OCT1 may participate in cell cycle regulation [94,95,96,97], while HSF2, SFMBT1, and NKX2 can modulate the expression of genes related to stress response, myogenesis, and embryonic cell differentiation [98,99,100].

**Figure 5:**
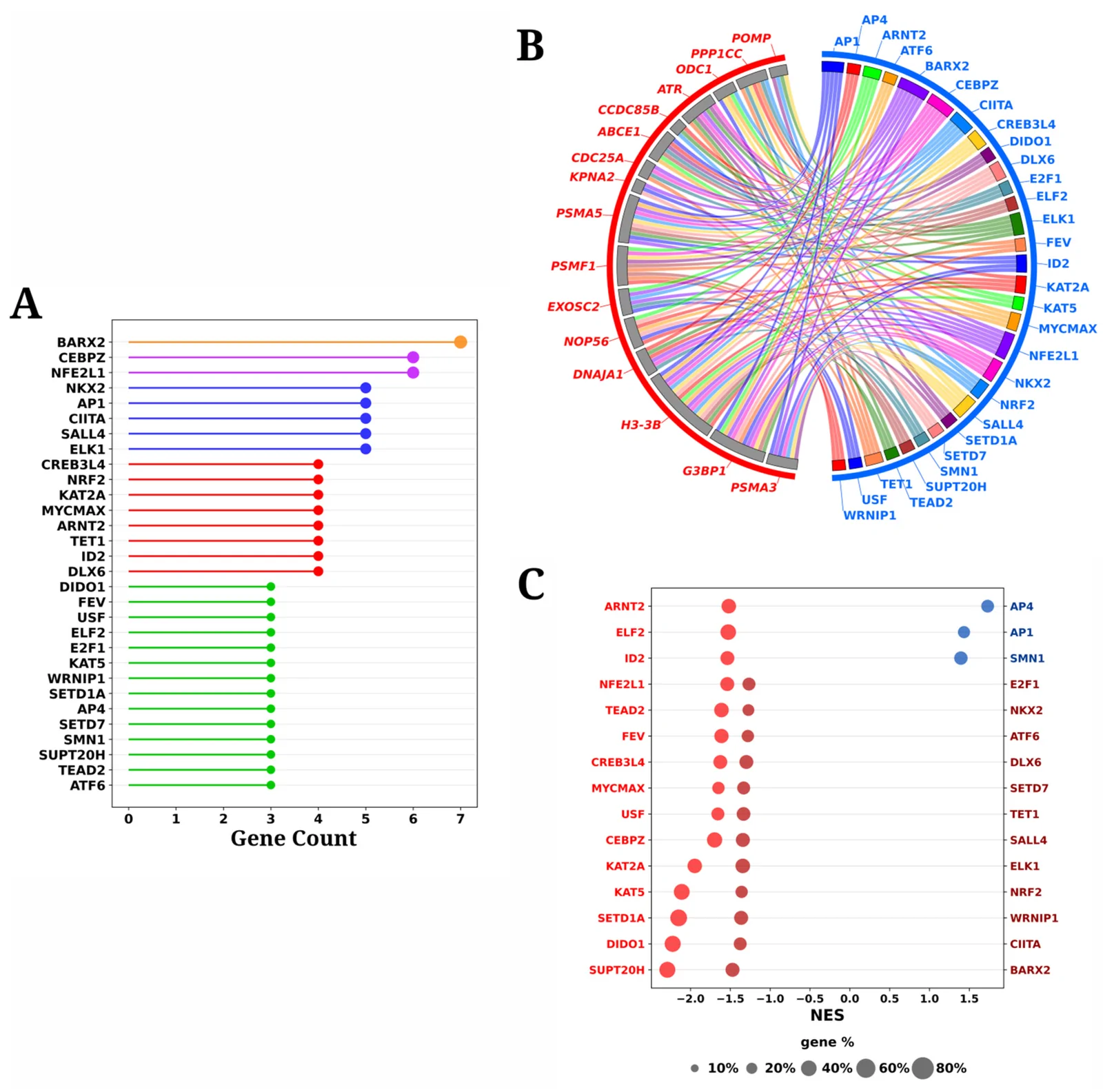
Examination of transcription factor (TF) activity associated with hub genes with increased expression in THP-1 HDC cells. (**A**) The quantity of hub genes regulated by transcription factors. (**B**) Association between discovered TFs (blue) and hub genes (red). (**C**) Gene Set Enrichment Analysis with identified transcription factors (colored dots represent gene collections; shading represents the axis on which each set is displayed). FDR ≤ 0.05.

Among the TFs associated with hub genes with decreased expression, specifically BARX2, CEBPZ, NFE2L1, NKX2, AP1, CIITA, SALL4, ELK1, CRED3L4, NRF2, KAT2A, MYCMAX, ARNT2, TET1, ID2, DLX6, DIDO1, FEV, USF, ELF2, E2F1, KAT5, WRNIP1, AP-4, SETD1A, SETD7, SMN1, and TEAD2, those interacting with three or more identified ISGs were selected (Figure 5A). Furthermore, «gene-TF» interactions were identified (Figure 5B), and a GSEA was performed, showing negative enrichment for 27 transcription factor sets, which may indicate a decrease in their activity. Three transcription factor sets (AP1, AP4, and SMN1) showed positive enrichment, which may indicate potential enhancement of their transcriptional activity (Figure 5C).

It was shown that the identified TFs, including BARX2, DLX6, TET1, SALL4, TEAD2, FEV, E2F1, MYCMAX, ELK1, WRNIP1, KAT5, AP1, AP4, and ELF2, serve as regulators of the cell cycle [101,102,103,104,105,106,107,108,109,110,111,112,113,114,115,116]. Transcription factors KAT2A and CIITA are involved in the regulation of the immune response [93,117], while transcription factors ATF6, NRF2, and NFE2L1 regulate cellular homeostasis [118,119,120]. In contrast, transcription factors BARX2, NKX2, CEBPZ, ARNT2, CRED3L4, DIDO1, SETD7, SMN1, and SUPT20H are involved in the regulation of actin cytoskeleton dynamics, expression of extracellular matrix components, apoptosis, stress-induced gene expression, protein synthesis, formation of neuromuscular synapses, and embryonic cell differentiation [100,101,121,122,123,124,125,126,127].

Thus, the results indicate that in apoptosis-resistant THP-1 cells, the identified TFs associated with hub ISGs showing increased expression are primarily involved in the regulation of pro-inflammatory interferon-associated signaling pathways. In contrast, the TFs associated with hub ISGs with decreased expression are mainly linked to the control of the cell cycle. The obtained data suggest that in apoptosis-resistant THP-1 cells under a pro-inflammatory microenvironment, there is an enhancement of the transcriptional activity of IFN-associated regulators accompanied by a weakening of the regulatory influence of factors controlling proliferation.

### 3.5. Comparison of Transcription Profiles of Apoptosis-Resistant THP-1 Cells in a Pro-Inflammatory Microenvironment and Clinical Samples of Refractory AMoL

Next, to validate our in vitro model of pro-inflammatory activation of AMoL cells and identify similar molecular changes in vivo, we performed a similar analysis on clinical transcriptomic data from patients with acute monocytic leukemia (AMoL). Since the THP-1 cell line is classified as acute monocytic leukemia (AMoL) according to the FAB classification and further characterized as AML with *KMT2A* translocation according to the WHO classification [128], transcriptomic data from 55 clinical samples of AMoL with characteristic t(9;11)(p21.3;q23.3) (*MLLT3-KMT2A* hybrid gene) and a mutation in the *NRAS* gene p.G12D were obtained from the beatAML2 project open database [32] and used. The obtained transcriptomic data were divided into two groups based on patient response to therapy: refractory (therapeutically resistant) AMoL, including 14 samples, and AMoL with achieved remission (complete response, therapeutically sensitive), including 41 samples. The analysis focused on signaling pathways related to interferons and transcription factors common between clinical samples and THP-1 HDC AMoL cells.

**Figure 6:**
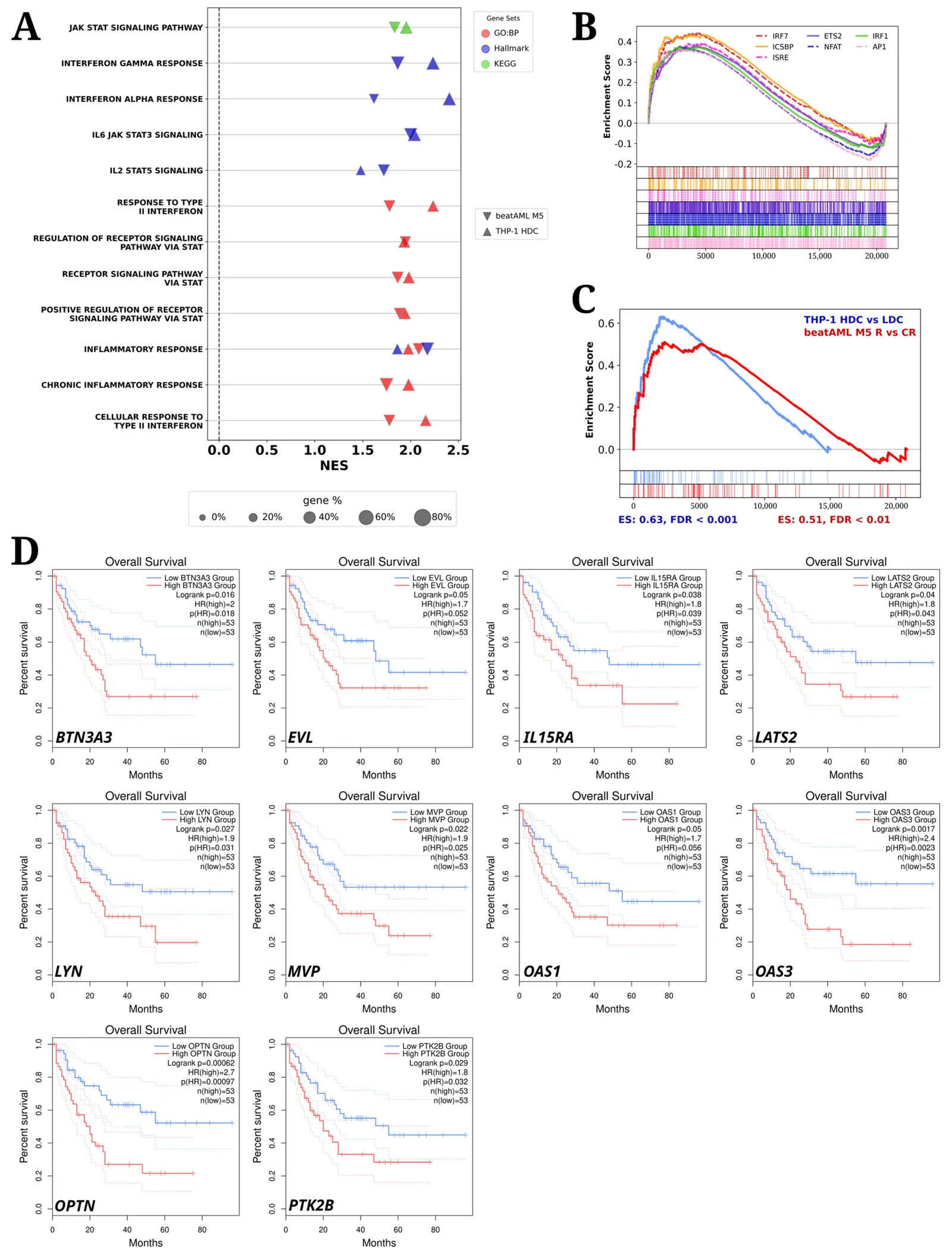
Comparison of transcription profiles of apoptosis-resistant THP-1 cells in a pro-inflammatory microenvironment and clinical samples of refractory AMoL. (**A**) Examination of interferon-related signaling pathways (SPs) in samples of refractory acute myeloblastic leukemia compared to THP-1 HDC using gene sets from the Hallmark, Gene Ontology: Biological Processes (GO:BP), and Kyoto Encyclopedia of Genes and Genomes (KEGG) collections obtained from MSigDB. (**B**) Result of GSEA of transcription factor activity in samples of refractory acute myeloblastic leukemia obtained from the Transcription Factor Targets (TFTs) collection of the MSigDB database. (**C**) Transcription activity of genes in IRDS molecular signatures in samples of refractory acute myeloblastic leukemia compared to THP-1 HDC. Results of GSEA are presented for FDR ≤ 0.05. (**D**) Analysis of patient survival depending on expression of identified ISGs. logrank *p* ≤ 0.05.

Analysis of differential gene expression based on RNA sequencing data from patients with AMoL and subsequent GSEA using the Hallmark (H), GO:BP, and Kyoto Encyclopedia of Genes and Genomes (KEGG) collections showed that refractory AMoL samples, as well as apoptosis-resistant THP-1 HDC cells, had statistically significant positive enrichment of gene sets including «JAK/STAT Signaling Pathway» (KEGG collection), «Inflammatory Response», «Regulation of Receptor Signaling Pathway via STAT», «Positive Regulation of Receptor Signaling Pathway via STAT», «Receptor Signaling Pathway via STAT», «Cellular Response to Type II Interferon», «Response to Type II Interferon», and «Chronic Inflammatory Response» (GO:BP collection), as well as «Inflammatory Response», «Interferon Gamma Response», and «Interferon Alpha Response» (Hallmark collection) compared to therapy-sensitive AMoL samples (Figure 6A).

Analysis of TFs associated with hub ISGs in refractory AMoL samples, as well as apoptosis-resistant THP-1 HDC cells, compared to therapy-sensitive AMoL samples showed statistically significant positive enrichment for IRF1, IRF7, ISRE, ICSBP, as well as NFAT, ETS2, and AP1 (Figure 6B), indicating the activation of these transcription factors in the studied samples. Additionally, we performed GSEA for apoptosis-resistant THP-1 cells and refractory AMoL samples using the Interferon-Related DNA Damage Resistance Signature (IRDS) gene set, a group of ISGs whose increased expression is found in several oncological diseases and associated with increased resistance to DNA-damaging agents and radiotherapy [35,36,129,130,131]. It was found that both in apoptosis-resistant THP-1 HDC cells and in refractory AMoL samples, the studied gene set showed statistically significant positive enrichment compared to the respective control groups (Figure 6C).

Next, a patient survival analysis was performed using the GEPIA service for clinical AMoL samples depending on the expression levels of ISGs identified in apoptosis-resistant THP-1 HDC cells. Results showed that increased expression levels of *IL15RA*, *BTN3A3*, *OAS1*, *OAS3*, *MVP*, *EVL*, *OPTN*, *PTK2B*, *LYN*, and *LATS2* were statistically associated with lower survival rates (log-rank *p* ≤ 0.05). This correlation indicates that the increased expression levels of genes observed in THP-1 HDC cells are associated with a poorer prognosis for patients (Figure 6D). It is important to note that there is a statistically significant correlation between elevated expression levels of identified ISGs and lower survival rates among patients with AMoL.

Thus, the results obtained indicate a high degree of correspondence between the data for apoptosis-resistant THP-1 cells in the model of a pro-inflammatory microenvironment and clinical samples of refractory AMoL, confirming the relevance and validity of the proposed in vitro model of pro-inflammatory activation of AML cells for studying mechanisms of therapeutic resistance. Furthermore, the results indicate a common interferon-associated stress-adaptive mechanism associated with resistance to DNA-damaging agents and radiotherapy in apoptosis-resistant THP-1 cells in the pro-inflammatory microenvironment model and clinical samples of refractory AMoL. Finally, the association of increased expression of ISGs identified in THP-1 HDC cells with poorer survival rates in patients with AMoL supports the clinical significance of this signaling axis and confirms that the molecular changes identified in our in vitro model reflect prognostically unfavorable processes *in vivo*.

## 4. Discussion

Our previous studies demonstrated that, in an in vitro model of a pro-inflammatory microenvironment in high-density cultures (HDCs) of THP-1 AMoL cells, pro-inflammatory activation is accompanied by the activation of interferon-associated signaling pathways in cells displaying the previously established resistance to the apoptosis-inducing effects of cytarabine, doxorubicin, and the cytokine TRAIL [20,26]. These results are consistent with, and confirm at the transcriptomic level, our previously reported findings that pro-inflammatory activation of AML cells in HDCs is associated with the transcriptional activation of type I and II interferon and NF-κB/STAT-dependent signaling pathways, including activation of the transcription factors IRFs and STATs [26]. Extending these prior observations, a key finding of the present study is the identification of a specific transcriptional profile in apoptosis-resistant THP-1 HDC cells, characterized by the activation of interferon signaling pathways, primarily of type II, as well as the involvement of transcription factors (TFs) such as IRF1, IRF7, and the ISRE complex and more. We further identified key interferon-stimulated genes (ISGs), e.g., OAS1, OAS3, LYN, and PTK2B. For a subset of these ISGs, a significant impact on patient survival was identified. Importantly, transcriptional activation was detected not only for TFs but also for gene sets of interferon signaling pathways, with activation observed in HDC cultures and in refractory patients compared with therapy-sensitive/complete response patients. Moreover, activation of the IRDS (Interferon-Related DNA Damage Resistance Signature) was identified in HDC and in refractory patients’ sample data.

Furthermore, a significant change in the expression of proteasome system components (*PSMA3*, *PSMA5*, *PSMB2*, *PSMF1*, *POMP*) was identified in apoptosis-resistant THP-1 HDC cells, suggesting a potential role of the pro-inflammatory microenvironment in reprogramming the proteasomal system in apoptosis-resistant THP-1 AMoL cells. The identified link between the inflammatory response and resistance to cytotoxic stimuli in THP-1 cell cultures suggests that the high-density microenvironment mimics pathological or near-pathological conditions of the tumor microenvironment in AMoL [6,12,13].

We hypothesize that the *in vitro* simulated pro-inflammatory microenvironment, in addition to activating the expression of anti-apoptotic proteins [20] and decreasing the transcriptional activity of cell cycle control genes [26], may stimulate the activation of interferon signaling in cells associated with impaired mitophagy and accumulation of dysfunctional mitochondria, as well as increased secretion of TNF-α [20,22]. Simultaneously, the activation of interferon signaling induced by the pro-inflammatory microenvironment may serve as an additional protective mechanism against cell death induced by antitumor agents and effectors of antitumor immunity, leading to the enhancement of pro-inflammatory survival signaling pathways and the formation of an adaptive phenotype characterized by suppressed mitophagy, remodeling of mitochondrial functions, and resistance to apoptosis [15,16,18,19,20,26]. Thus, interferons are capable of stimulating non-canonical signaling pathways PI3K/Akt/mTOR, MAPK, and RAS, which regulate the cell cycle and survival [12,13,132], as well as enhancing the NF-κB signaling pathway [133,134,135], thereby exerting an indirect influence on the increased expression of anti-apoptotic proteins.

An important result of the study is also the comparison between pro-inflammatory activation in an in vitro model of a pro-inflammatory microenvironment and LPS-induced activation of cells, which showed that both conditions induce interferon signaling compared to the control (LDC). The LPS condition exhibits a more pronounced increase in the content of Irf9 and Stat2 proteins, as well as pStat2, which is likely due to the activation of Type I interferon signaling pathways resulting from TLR4-MyD88-NF-κB-mediated signaling [53,136,137]. Canonical LPS-induced activation of THP-1 cells is mediated through the expression of IFN-β, which specifically stimulates the activation of Stat1/2 and the formation of the Stat1/2-Irf9 (ISGF3) complex, which translocates to the nucleus and binds to ISRE DNA sequences, stimulating the expression of ISGs [53,54,55]. At the same time, lower levels of Stat1 and its phosphorylated forms in THP-1 LPS cells compared to THP-1 HDC cells are likely associated with pre-incubation in the presence of LPS, which reduces Stat1 phosphorylation [136,138]. In this context, under conditions of Stat1 deficiency within the classic ISGF3 heterotrimer, its function may be partially compensated by the Stat2-Irf9 complex, which is capable of providing a similar profile of transcriptional activity for interferon-stimulated genes [139,140,141]. A different pattern of STAT pathway activation is observed in THP-1 HDC cells, presumably mediated by IFN-γ [53,136,142]. We suggest that in the case of THP-1 HDC cells, Stat1 phosphorylation leads to the formation of a Stat1 homodimer (GAF), which binds to gamma-activated site (GAS) sequences, activating ISG transcription [52,143]. The results obtained demonstrate a fundamentally different mechanism of STAT pathway activation under different conditions of pro-inflammatory activation.

We also observed a decrease in the surface expression of the IFNAR1 receptor while IFNAR2 and IFNGR1/2 were maintained. The decrease in surface expression of the IFNAR1 receptor may be associated with the formation of negative feedback loops and increased expression and potential accumulation of SOCS proteins (Suppressor of Cytokine Signaling). SOCS-1/3 proteins are induced via the STAT-dependent pathway and inhibit JAK kinases, activating ubiquitin-dependent degradation of the receptor complex [144,145]. In turn, upon binding of Type I or Type II IFNs, activated JAK/TYK kinases can phosphorylate serine residues of the receptors, which recruits the E3 ligase SCFβ-TrCP and leads to ubiquitination and endocytosis of the receptors into lysosomal compartments, serving as a negative feedback mechanism [146,147]. Additionally, pro-inflammatory cytokines (IL-1β, TNF-α) can phosphorylate IFNRs via p38 MAPK, causing ligand-independent internalization of the receptors [148]. Further analysis of the transcriptional profile of apoptosis-resistant THP-1 HDC cells compared to apoptosis-sensitive THP-1 LDC cells allowed for the identification of complex transcriptional reprogramming induced by the pro-inflammatory microenvironment. Specifically, an increase in the expression of interferon-induced genes regulated by transcription factors of the IRF family (IRF1, IRF7, IRF8) and STAT (STAT5A/B), among others, was shown, with a simultaneous decrease in the transcriptional activity of genes regulated by cell cycle control transcription factors (E2F1, MYCMAX, etc.) [20,21,30]. The identified functional divergence between TFs regulating hub ISGs with increased expression (e.g., IRF1, IRF7, STAT5, and others) associated with pro-inflammatory and interferon-mediated signaling; and TFs regulating the expression of ISGs with reduced expression (e.g., E2F1, MYC, BARX2, and others) associated with cell cycle control, suggests that cells under HDC conditions undergo a transcriptional transition: suppressing mechanisms of normal cell cycle and homeostasis maintenance (via decreased activity of TFs such as MYCMAX or E2F1) while activating interferon-mediated cell survival programs. The data obtained regarding the activation of STAT pathways and IRF transcription factors in the context of the role of the pro-inflammatory microenvironment in AMoL drug resistance are consistent with findings from studies demonstrating the role of these signaling cascades in increasing the resistance of myeloid cells to chemotherapeutic agents [149,150,151]. Thus, the results presented in this work, in combination with previously published studies, suggest that an in vitro model of a «pro-inflammatory microenvironment» in THP-1 cells leads to the formation of a phenotype promoting survival in response to cytotoxic stimuli and likely supports research describing the plasticity of AML cells [152,153,154,155,156,157,158].

An interesting result was the observed decrease in the expression of proteasome system components, specifically *PSMA3*, *PSMA5*, *PSMB2*, *PSMF1*, and *POMP*. Although this phenomenon requires further detailed study, it may indicate the formation of a «vicious cycle» of pro-inflammatory activation: a deficiency in functional proteasomes may lead to protein accumulation and subsequent increase in levels of inflammatory proteins such as NF-κB p65, which enhances pro-inflammatory activity [20,159,160,161]. It is important to note that literature data indicate that changes in proteasome functions can trigger the activation of the unfolded protein response (UPR), which may further stimulate interferon-induced genes in various cell types and tissues, thereby enhancing pro-inflammatory signaling [88,162,163,164]. However, the cause of this proteasomal dysfunction remains unclear. The transformation of cells under HDC conditions may also be accompanied by changes in the expression of HLA molecules (*HLA-A*, *HLA-B*, *HLA-E*). It can be suggested that, against the background of decreased transcriptional activity of «classical» proteasome genes, a transition to immunoproteasomes may occur, which is indirectly indicated by changes in the expression of *HLA* molecules (*HLA-A*, *HLA-B*, *HLA-E*) [165,166].

Notably, the profile of transcription factors associated with hub interferon-stimulated genes (ISGs) in samples of refractory AMoL reproduced the corresponding pattern of apoptosis-resistant THP-1 HDC cells. Increased activity of IRF1, IRF7, ISRE, and ICSBP identified during transcription factor enrichment indicates coordinated activation of regulatory interferon signaling in both systems. IRF1 and IRF7 are central activators of Type I and Type II interferon gene transcription; their combined activation provides a self-sustaining loop mechanism that enhances the expression of ISGs [167,168]. Concurrent enrichment of NFAT, ETS2, and AP1 motifs further indicates a broad convergence of inflammatory transcription programs [169,170,171].

A key result of the present study is the demonstration that the Interferon-Related DNA Damage Resistance Signature (IRDS) gene set—a group of ISGs whose overexpression is associated with resistance to DNA-damaging agents and radiotherapy in several oncological conditions—showed statistically significant positive enrichment in both apoptosis-resistant THP-1 HDC cells and refractory AMoL samples compared to the respective control groups. Published data indicate that the IRDS gene pattern, including genes such as *STAT1*, *IRF7*, the *OAS* family, and *BST2*, constitutively suppresses pro-apoptotic programs and promotes enhanced DNA damage repair, thereby reducing the efficacy of standard cytostatic regimens. This is particularly significant in the context of AMoL with *KMT2A* rearrangement, characterized by an initially aggressive clinical course and a high frequency of resistance to standard chemotherapy [35,36,172,173].

Furthermore, a survival analysis conducted using the GEPIA platform based on clinical data from patients with AMoL established a statistically significant inverse correlation between increased expression of ISGs identified in THP-1 HDC cells—*IL15RA*, *BTN3A3*, *OAS1*, *OAS3*, *MVP*, *EVL*, *OPTN*, *PTK2B*, *LYN*, and *LATS2*—and overall survival (log-rank *p* ≤ 0.05). In this regard, the clinical significance of the association between prognostically unfavorable ISGs identified in the THP-1 HDC model and reduced survival in patients with AMoL highlights the potential of these genes as promising prognostic biomarkers and therapeutic targets in refractory AMoL.

Collectively, the obtained data demonstrate a high degree of molecular congruence between the transcriptome landscape of apoptosis-resistant THP-1 cells in an *in vitro* model of a pro-inflammatory microenvironment and clinical samples of refractory AMoL. This correspondence encompasses three independent levels of molecular organization: the enrichment of canonical STAT signaling pathways and interferon responses across multiple annotation collections; the activation of a general group of transcription factors forming the core of the interferon regulatory apparatus (IRF1, IRF7, ISRE/ICSBP); and the positive enrichment of the IRDS signature associated with resistance to DNA-damaging therapy. Such structural convergence of patterns indicates that the proposed in vitro model can replicate pro-inflammatory interferon-associated molecular mechanisms of resistance clinically relevant to the refractory course of AMoL.

Although the HDC model does not reconstruct the bone marrow niche, we use «functional biomimetic» to describe its selective emulation of leukemia-cell-derived inflammatory adaptation and associated drug resistance. It lacks a hydrogel matrix, mesenchymal stromal cells, endothelial cells, microfluidic perfusion, and controlled oxygen gradients. Nevertheless, it recreates hypercellularity, aseptic auto-/paracrine inflammatory activation [20,26] and a reversible, survival-promoting, drug-resistant state of leukemic cells [21,30]. Despite this minimalism, the model is well suited to investigating the molecular mechanisms of resistance that leukemia cells generate autonomously, independently of stromal, endothelial, and other niche components present in the complex bone marrow microenvironment *in vivo* [174,175,176,177].

HDC cells autonomously remodel their soluble microenvironment, as reflected in increased concentrations of several cytokines, chemokines, and growth factors in the culture medium—including IL-1β, IL-1ra, IL-6, IL-9, TNF-α, MCP-1, MIP-1β, RANTES, bFGF, G-CSF, IP-10, and MIP-1α [20]. This pattern is consistent with the dysregulated cytokine network and chronic inflammatory signaling reported in AML bone marrow [12,13,178,179,180]. In addition, HDC cells exhibit activation of interferon-associated signaling [26], which the present findings extend by demonstrating its association with the resistant phenotype. The clinical literature indicates context-dependent roles of interferon signaling in AML, with type I interferon signaling in malignant blasts linked to treatment efficacy and increased IFNγ signaling associated with treatment resistance and adverse outcomes, particularly in monocytic AML [15,16,17,18,19,149,181,182].

By restricting the system to leukemia cells, the reductionist design enables investigation of leukemia-cell-derived inflammatory adaptation and homotypic interactions separately from complex stromal and matrix-mediated effects. Because HDC cells autonomously develop a pro-inflammatory and interferon-associated activation state, the model also offers an opportunity to dissect how this activation arises, is sustained through auto-/paracrine loops, and contributes to cellular drug resistance. In particular, it allows controlled perturbation of inflammatory cytokines, interferon-associated pathways, or their downstream mediators to test whether such signals are sufficient or necessary for the drug-resistant/drug-tolerant phenotype, and whether resistance can be reversed by interrupting these circuits. This provides a focused, hypothesis-generating platform for separating leukemia-cell-intrinsic resistance mechanisms from niche-dependent protection, before validating candidate mechanisms in primary samples or more complex bone marrow-like models.

At the same time, the present implementation has clear limitations: it relies on a single immortalized cell line and does not reproduce cell-matrix interactions or heterotypic crosstalk with stromal, endothelial, and immune cells. Therefore, it cannot fully reflect the complexity of the bone marrow niche.

Overall, the HDC model provides an experimentally tractable and reproducible system for investigating leukemia-cell-derived inflammatory adaptation and associated resistance. Incorporating selected niche components and validating the findings in primary samples could help assess its disease relevance; exploratory compound testing remains a prospective application requiring assay-specific validation.

## 5. Conclusions

The present study demonstrated that conditions of pro-inflammatory activation, mimicked by the *in vitro* high-density culture model of THP-1 cells, could be associated with the formation of a stress-adaptive phenotype in AMoL cells resistant to cytotoxic agents. Based on the observed activation of interferon signaling pathways, we hypothesize that interferon-associated signaling contributes to the formation or maintenance of this phenotype. Activation of JAK/STAT signaling, interferon-induced transcription factors (IRF1, IRF7, IRF8, STATs), and hub ISGs (e.g., *OAS1*, *OAS3*, *LYN*, *PTK2B*) is coupled with the suppression of cell cycle control transcriptional programs and a decrease in the expression of proteasome system components, indicating profound transcriptional and metabolic remodeling toward survival and resistance to apoptosis.

Comparison of LPS-induced activation revealed fundamentally different modes of interferon signaling activation, highlighting the critical importance of the microenvironment’s nature for the selection of survival programs in leukemia cells. The replication of key signatures (JAK/STAT activation, IRF factors, interferon-stimulated genes) in clinical samples of refractory AMoL subtype M5 and their correlation with poorer patient survival outcomes confirm the biological and clinical relevance of our proposed in vitro model.

Collectively, our data support the concept that drug resistance in AMoL may arise through the activation of evolutionarily conserved antiviral and interferon-associated cascades under conditions of hypercellularity and a pro-inflammatory microenvironment. The HDC model selectively reproduces this leukemia-cell-derived inflammatory adaptation and its associated resistant phenotype, providing an experimentally tractable system for dissecting these mechanisms. The identified hub ISGs, features of proteasomal dysfunction, and transcription factor networks (IRF, STAT, BCL6, NF-κB) represent promising targets for further mechanistic studies aimed at overcoming resistance and improving therapeutic outcomes in AMoL.

## Figures and Tables

**Table 1:** DNA oligonucleotide primers for qRT-PCR used in the study.

Gene	Forward/Reverse	Sequence
NM_000619 (*IFNG*)	Forward	GTTTTGGGTTCTCTTGGCTG
	Reverse	ACACTCTTTTGGATGCTCTGG
NM_002176 (*IFNB1*)	Forward	AGTAGGCGACACTGTTCGTG
	Reverse	ATAGATGGTCAATGCGGCGT
NM_021068 (*IFNA4*)	Forward	GGAGGTTGTCAGAGCAGAAATC
	Reverse	GTCATCTGTAAAGGACTAGTGCC
NM_002171 (*IFNA10*)	Forward	CTCACTTCTATAACCACGACGC
	Reverse	CCTGCACAGGTATACACGACA
NM_021057.2 (*IFNA7*)	Forward	GACTTCATCCTGGCTGTGAG
	Reverse	TTGTGGTGGTTATAGAAGTGAGTC
NM_002172 (*IFNA14*)	Forward	TCTTCGGGATTCCCAATGGC
	Reverse	ATTACAGCCCAGAGAGCAGC
NM_002173 (*IFNA16*)	Forward	TTGGGGTGGAAGAGATTGCC
	Reverse	TCTTCCCCATCAGATAAAGAGTGA
NM_021268 (*IFNA17*)	Forward	GTTACCCATCTCAAGTAGCCTAG
	Reverse	CAGATCACAGCCTAGAGAACAG
NM_002175 (*IFNA21*)	Forward	CTGAATGACCTGGAAGCCTG
	Reverse	GGCAGAAGTCATAGAAGTGTGG

## Data Availability

The RNA-seq data for the cell lines described in this study are available through the Gene Expression Omnibus (GEO) database under accession number GSE298747 or via the direct link: https://www.ncbi.nlm.nih.gov/geo/query/acc.cgi?acc=GSE298747 (accessed on 2 June 2025). The RNA-seq data for patients with Acute Myeloid Leukemia is available from the original article at DOI: 10.1016/j.ccell.2022.07.002 or via the direct repository link: https://biodev.github.io/BeatAML2/ (accessed on 20 February 2026).

## Supplementary Materials

The following supporting information can be downloaded at https://www.mdpi.com/article/10.3390/biology15201876/s1, Table S1: MultiQC general statistics reads quality report; Figure S1: Quality control of sequencing libraries; Table S2: Interferon-Related DNA-Damage Signature (IRDS) gene set for GSEA processing; Figure S2: Representative Western blot membranes of STAT1, STAT2, and IRF9 protein expression in human THP-1 AMoL cells; Table S3: Specific chromosomal aberrations characteristic of THP-1 cells, identified by the aCGH method; Table S4: Concentration of IFNγ and IFN-stimulated chemokines in the culture medium of THP-1 LDC, THP-1 HDC and THP-1 LPS cell; Figure S3: Cytotoxic effects of TRAIL, doxorubicin, and cytarabine on acute myeloid leukemia cells cultured at low (LDC) or high (HDC) density cultures; Figure S4: Interferon receptors surface expression and transcriptional JAK/STAT signaling pathway activation; Figure S5: Full protein–protein interaction network of identified interferon-stimulated genes.

## Abbreviations

AML	Acute Myeloid Leukemia
AMoL	Acute Monocytic Leukemia
DGE	Differential Gene Expression
FDR	False Discovery Rate
GSEA	Gene Set Enrichment Analysis
GO:BP	GeneOnthology: Biological Processes
HDC	High-Density Culture
IFN	Interferon
ISG	Interferon-Stimulated Gene
LDC	Low-Density Culture
LPS	Lipopolysaccharide
MHC	Major Histocompatibility Complex
MSigDB	Molecular Signature DataBase
NES	Normalized Enrichment Score
PPI	Protein–Protein Interaction
SP	Signal Pathway
TF	Transcription Factor
TFT	Transcription Factor Target
WP	WikiPathways
