# Supplementary material for: A Biomimetic In Vitro Model of the Pro-Inflammatory Microenvironment Recapitulates Type I/II Interferon Signaling and Refractory Acute Monocytic Leukemia
Source: Biology (Basel). 2026 Oct 17;15(20):1876. doi: 10.3390/biology15201876 (PMC13701761; doi:10.3390/biology15201876)
Supplement: Supplementary file 1 [file biology-15-01876-s001.zip › biology-4574401-supplementary.pdf]

Supplementary materials

Table S1. MultiQC general statistics reads quality report. LDC – Low-Density Culture; HDC – High-Density Culture.

Sample Name	Average Read Length, bp	Median Read Length, bp	M Seqs
THP-1HDC1	50	50	11.2
THP-1HDC2	50	50	10.6
THP-1HDC3	50	50	10.8
THP-1LDC1	50	50	10.1
THP-1LDC2	50	50	11.5
THP-1LDC3	50	50	10.6

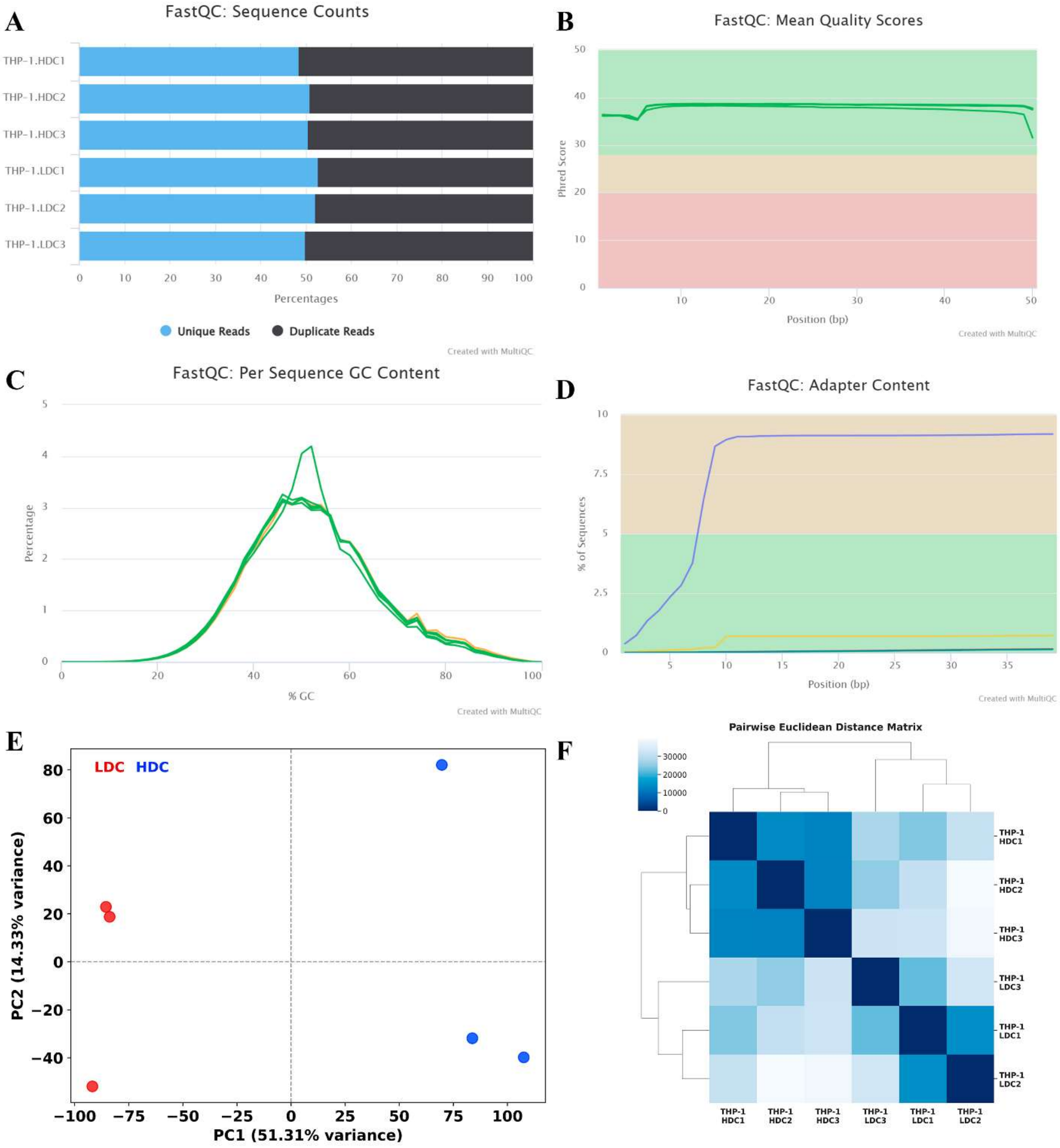

Figure S1. Quality control of sequencing libraries. (A) Unique and duplicate sequence count distributions per sample. (B) Mean Phred quality score per sequencing sample. (C) GC content profiles per sample. (D) Adapter contamination rates per sample. E) Principal Component Analysis (PCA) and (F) Sample-to-sample Euclidean distance matrix calculated using all differentially expressed genes. LDC - Low-Density Culture; HDC - High-Density Culture.

Table S2. Interferon-Related DNA-Damage Signature (IRDS) gene set for GSEA processing.

#	**gene**	#	**gene**	#	**gene**	#	**gene**
**1**	*ADAR*	**25**	*G1P2*	**49**	*IFITM3*	**73**	*PLSCR1*
**2**	*ALDH3A1*	**26**	*G1P3*	**50**	*IGF2*	**74**	*PML*
**3**	*APOBEC3A*	**27**	*GALC*	**51**	*IRF1*	**75**	*RAP2C*
**4**	*APOBEC3B*	**28**	*GBP1*	**52**	*IRF7*	**76**	*ROBO1*
**5**	*APOBEC3C*	**29**	*GBP2*	**53**	*ISG15*	**77**	*RSAD2*
**6**	*APOBEC3D*	**30**	*HERC6*	**54**	*ISG20*	**78**	*RTP4*
**7**	*APOBEC3F*	**31**	*HLA-B*	**55**	*LAMP3*	**79**	*SERPINB2*
**8**	*APOBEC3G*	**32**	*HLA-G*	**56**	*LGALS3BP*	**80**	*SH3YL1*
**9**	*APOBEC3H*	**33**	*HPSE*	**57**	*LY6E*	**81**	*SLC15A3*
**10**	*BST2*	**34**	*HSD17B1*	**58**	*MAP3K14*	**82**	*SLC25A28*
**11**	*CA2*	**35**	*IFI1H*	**59**	*MB21D1*	**83**	*SLC6A15*
**12**	*CCNA1*	**36**	*IFI27*	**60**	*MCL1*	**84**	*SSBP3*
**13**	*CD59*	**37**	*IFI35*	**61**	*MOV10*	**85**	*STAT1*
**14**	*CD74*	**38**	*IFI44*	**62**	*MS4A4A*	**86**	*SUN2*
**15**	*CXCL1*	**39**	*IFI44L*	**63**	*MX1*	**87**	*THBS1*
**16**	*CXCL10*	**40**	*IFI6*	**64**	*MX2*	**88**	*TIMP3*
**17**	*DAZ1*	**41**	*IFI6*	**65**	*NAMPT*	**89**	*TREX1*
**18**	*DCN*	**42**	*IFIH1*	**66**	*NT5C3A*	**90**	*TRIM14*
**19**	*DDIT4*	**43**	*IFIT1*	**67**	*NT5C3B*	**91**	*TRIM25*
**20**	*DDX58*	**44**	*IFIT2*	**68**	*OAS1*	**92**	*TRIM5*
**21**	*DDX60*	**45**	*IFIT3*	**69**	*OAS2*	**93**	*TncRNA*
**22**	*EIF2AK2*	**46**	*IFIT5*	**70**	*OAS3*	**94**	*USP18*
**23**	*FLJ20035*	**47**	*IFITM1*	**71**	*OASL*	**95**	*ZC3HAV1*
**24**	*FLJ38348*	**48**	*IFITM2*	**72**	*P2RY6*	**96**	*ZNF273*

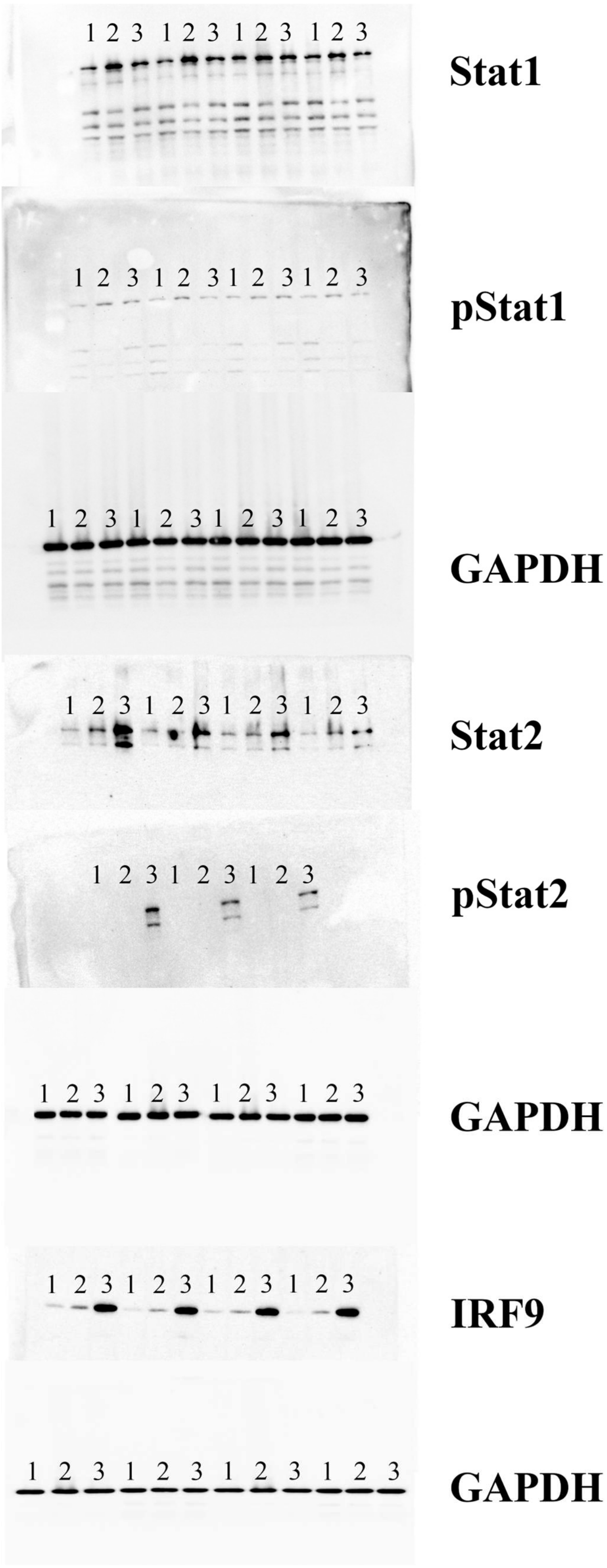

Figure S2. Representative Western blot membranes of STAT1, STAT2, and IRF9 protein expression in human THP-1 AMoL cells. Cells were cultured as low-density culture (THP-1 LDC, 1), high-density culture (THP-1 HDC, 2), or stimulated with lipopolysaccharide (THP-1 LPS, 3).

Table S3. Specific chromosomal aberrations characteristic of THP-1 cells, identified by the aCGH method.

Chr	Chr. aberration	Aberration
chr1	del(1)(p36.33p34.3)	1p36.33p34.3(746608_39881672)x1
	dup(1)(p34.3p34.2)	1p34.3p34.2(39971733_41310890)x10
	dup(1)(p34.2p34.1)	1p34.2p34.1(41310891_44247548)x3
	dup(1)(p32.3)	1p32.3(53884702_54829205)x3
	del(1)(p32.3p31.1)	1p32.3p31.1(55504656_75813195)x1
	del(1)(p31.1)	1p31.1(76965332_77422513)x1
	del(1)(p31.1p22.2)	1p31.1p22.2(81720872_90976359)x1
	dup(1)(p21.1p13.3)	1p21.1p13.3(107134686_121330906)x3
	dup(1)(q21.1q21.3)	1q21.1q21.3(144912461_154654367)x3
	dup(1)(q21.3q22)	1q21.3q22(154654368_155009170)x4
	dup(1)(q22)	1q22(155009171_156462310)x3
	dup(1)(q23.3)	1q23.3(160980382_161898826)x3
	dup(1)(q41q44)	1q41q44(217103290_247911730)x3
	dup(1)(q44)	1q44(248901720_249212668)x3
chr2	dup(2)(p15)	2p15(63352057_637444992)x1~2
chr3	del(3)(p26.3)	3p26.3(1153789_1242477)x1
	del(3)(q25.32)	3q25.32(157883142_158157989)x1
chr4	dup(4)(p16.1)	4p16.1(9371016_9476257)x3~4
	del(4)(q34.3)	4q34.3(182099817_182376978)x0
	del(4)(q34.3)	4q34.3(182403913_182500838)x1
	del(4)(q35.2)	4q35.2(188106143_188238599)x1
chr5	dup(5)(p15.33)	5p15.33(22149_2212048)x3
	dup(5)(p15.33p15.2)	5p15.33p15.2(2212049_14246208)x4
	dup(5)(p15.2p11)	5p15.2p11(14246209_46365277)x3
	dup(5)(q34)	5q34(161908454_162186029)x3
chr6	del(6)(p25.3p21.33)	6p25.3p21.33(163083_31480336)x1
	dup(6)(q21q27)	6q21q27(107814067_170921089)x3
chr7	del(7)(q31.1)	7q31.1(110814652_110905009)x1
chr8	dup(8)(pter->qter)	(8)x3
chr9	dup(9)(p24.3p23)	9p24.3p23(211086_12709898)x3
	dup(9)(p23p22.3)	9p23p22.3(12709899_15433696)x4
	dup(9)(p22.3p21.3)	9p22.3p21.3(15433697_20365833)x3
	del(9)(p21.3)	9p21.3(21289842_22241139)x0
	dup(9)(p21.3)	9p21.3(22818496_23813113)x3
	hmz(9)(p21.3p13.2)	9p21.3p13.2(22945119_37616445)x2 hmz
	dup(9)(p21.1p13.3)	9p21.1p13.3(33154643_34807568)x3
	dup(9)(p13.2p13.1)	9p13.2p13.1(37505349_40771232)x3
	dup(9)(q13q21.11)	9q13q21.11(68508510_71016040)x3~4
	hmz(9)(q21.11q34.13)	9q21.11q34.13(71051367_135893990)x2 hmz
	dup(9)(q34.2q34.3)	9q34.2q34.3(136828326_141018984)x3
chr10	hmz(10)(p15.1p11.21)	10p15.1p11.21(5389223_37722472)x2 hmz
	hmz(10)(q11.21q26.3)	10q11.21q26.3(42491351_133351434)x2 hmz
	del(10)(q23.31)	10q23.31(89664975_89693552)x0
	del(10)(q23.31)	10q23.31(89693553_89722948)x1
chr11	dup(11)(q23.3q25)	11q23.3q25(118374043_134934196)x3
chr12	del(12)(p13.33p11.1)	12p13.33p11.1(162848_34827047)x1
	del(12)(q11q13.12)	12q11q13.12(38153138_51183876)x1
chr13	hmz(13)(q12.11q34)	13q12.11q34 (19584477_115064542)x2 hmz
chr14	del(14)(q21.1q21.2)	14q21.1q21.2(43222699_43671873)x1

	del(14)(q24.3)	14q24.3(76149538_76303222)x1
chr17	del(17)(p13.3p11.2)	17p13.3p11.2(53449_21501243)x1
	dup(17)(q25.3)	17q25.3(78952180_79120623)x3
chr20	dup(20)(p13)	20p13(60747_481619)x2~3
	del(20)(p13p11.23)	20p13p11.23(512460_20788969)x1
	dup(20)(p11.22p11.21)	20p11.22p11.21(21367861_22628267)x3
chrX	del(X)(p11.4p11.3)	Xp11.4p11.3(41747131_44916537)x0
	del(X)(q28)	Xq28(153541230_153601836)x0
chrY	del(Y)(p11.31)	Yp11.31(2654831_2655026)x0
	del(Y)(p11.31)	Yp11.31(2656104_2878761)x0
	del(Y)(p11.2)	Yp11.2(6027028_9882201)x0
	del(Y)(q11.21q11.23)	Yq11.21q11.23(13872502_28644194)x0

Table S4. Concentration of IFNγ and IFN-stimulated chemokines in the culture medium of THP-1 LDC, THP-1 HDC and THP-1 LPS cell. Concentrations normalized to $1{\times}10^4$ cells. LDC – Low-Density Culture; HDC – High-Density Culture: LPS – Lipopolysaccharide.

#	Chemokine	Concentration, pg/ml	Sample	log10(Concentration), pg/ml
**0**	IP-10	0.11	THP-1 LDC	-0.96
**1**	IP-10	0.1	THP-1 LDC	-1.01
**2**	IP-10	0.08	THP-1 LDC	-1.11
**3**	IP-10	0.14	THP-1 LDC	-0.84
**4**	IP-10	773.78	THP-1 HDC	2.89
**5**	IP-10	708.48	THP-1 HDC	2.85
**6**	IP-10	665.46	THP-1 HDC	2.82
**7**	IP-10	652.41	THP-1 HDC	2.81
**8**	IP-10	1884.23	THP-1 LPS	3.28
**9**	IP-10	1924.92	THP-1 LPS	3.28
**10**	IP-10	1620.15	THP-1 LPS	3.21
**11**	IP-10	2007.45	THP-1 LPS	3.3
**12**	MIP-1α	0.1	THP-1 LDC	-1.02
**13**	MIP-1α	0.09	THP-1 LDC	-1.05
**14**	MIP-1α	0.09	THP-1 LDC	-1.04
**15**	MIP-1α	0.11	THP-1 LDC	-0.96
**16**	MIP-1α	3.1	THP-1 HDC	0.49
**17**	MIP-1α	2.59	THP-1 HDC	0.41
**18**	MIP-1α	2.72	THP-1 HDC	0.43
**19**	MIP-1α	3.03	THP-1 HDC	0.48
**20**	MIP-1α	37.25	THP-1 LPS	1.57
**21**	MIP-1α	30.45	THP-1 LPS	1.48
**22**	MIP-1α	27.68	THP-1 LPS	1.44
**23**	MIP-1α	28.53	THP-1 LPS	1.46
**24**	MIP-1β	1.51	THP-1 LDC	0.18
**25**	MIP-1β	2.33	THP-1 LDC	0.37
**26**	MIP-1β	1.96	THP-1 LDC	0.29
**27**	MIP-1β	2.18	THP-1 LDC	0.34
**28**	MIP-1β	416.41	THP-1 HDC	2.62
**29**	MIP-1β	376.68	THP-1 HDC	2.58
**30**	MIP-1β	445.88	THP-1 HDC	2.65
**31**	MIP-1β	394.01	THP-1 HDC	2.6
**32**	MIP-1β	766.19	THP-1 LPS	2.88
**33**	MIP-1β	753.05	THP-1 LPS	2.88
**34**	MIP-1β	708.75	THP-1 LPS	2.85
**35**	MIP-1β	746.47	THP-1 LPS	2.87
**36**	Rantes	40.9	THP-1 LDC	1.61
**37**	Rantes	33.03	THP-1 LDC	1.52
**38**	Rantes	37.53	THP-1 LDC	1.57

**39**	Rantes	42.95	THP-1 LDC	1.63
**40**	Rantes	569.93	THP-1 HDC	2.76
**41**	Rantes	525.84	THP-1 HDC	2.72
**42**	Rantes	585.95	THP-1 HDC	2.77
**43**	Rantes	566.1	THP-1 HDC	2.75
**44**	Rantes	149.25	THP-1 LPS	2.17
**45**	Rantes	150.45	THP-1 LPS	2.18
**46**	Rantes	170.06	THP-1 LPS	2.23
**47**	Rantes	166.38	THP-1 LPS	2.22
**48**	MCP-1	0.63	THP-1 LDC	-0.2
**49**	MCP-1	0.62	THP-1 LDC	-0.21
**50**	MCP-1	0.46	THP-1 LDC	-0.33
**51**	MCP-1	0.44	THP-1 LDC	-0.35
**52**	MCP-1	30.67	THP-1 HDC	1.49
**53**	MCP-1	31.19	THP-1 HDC	1.49
**54**	MCP-1	29.54	THP-1 HDC	1.47
**55**	MCP-1	32.86	THP-1 HDC	1.52
**56**	MCP-1	3681.72	THP-1 LPS	3.57
**57**	MCP-1	5192.1	THP-1 LPS	3.72
**58**	MCP-1	4719.63	THP-1 LPS	3.67
**59**	MCP-1	3670.31	THP-1 LPS	3.56
**60**	IFNγ	0.539	THP-1 LDC	-0.268411234813261
**61**	IFNγ	0.676	THP-1 LDC	-0.170053304058364
**62**	IFNγ	0.554	THP-1 LDC	-0.25649023527157
**63**	IFNγ	0.437	THP-1 LDC	-0.359518563029578
**64**	IFNγ	0.374	THP-1 HDC	-0.42712839779952
**65**	IFNγ	0.415	THP-1 HDC	-0.381951903287907
**66**	IFNγ	0.406	THP-1 HDC	-0.391473966422806
**67**	IFNγ	0.414	THP-1 HDC	-0.382999658879101
**68**	IFNγ	7.551	THP-1 LPS	0.878004470268025
**69**	IFNγ	7.312	THP-1 LPS	0.864036182725775
**70**	IFNγ	7.198	THP-1 LPS	0.857211842316892
**71**	IFNγ	7.303	THP-1 LPS	0.86350130064145

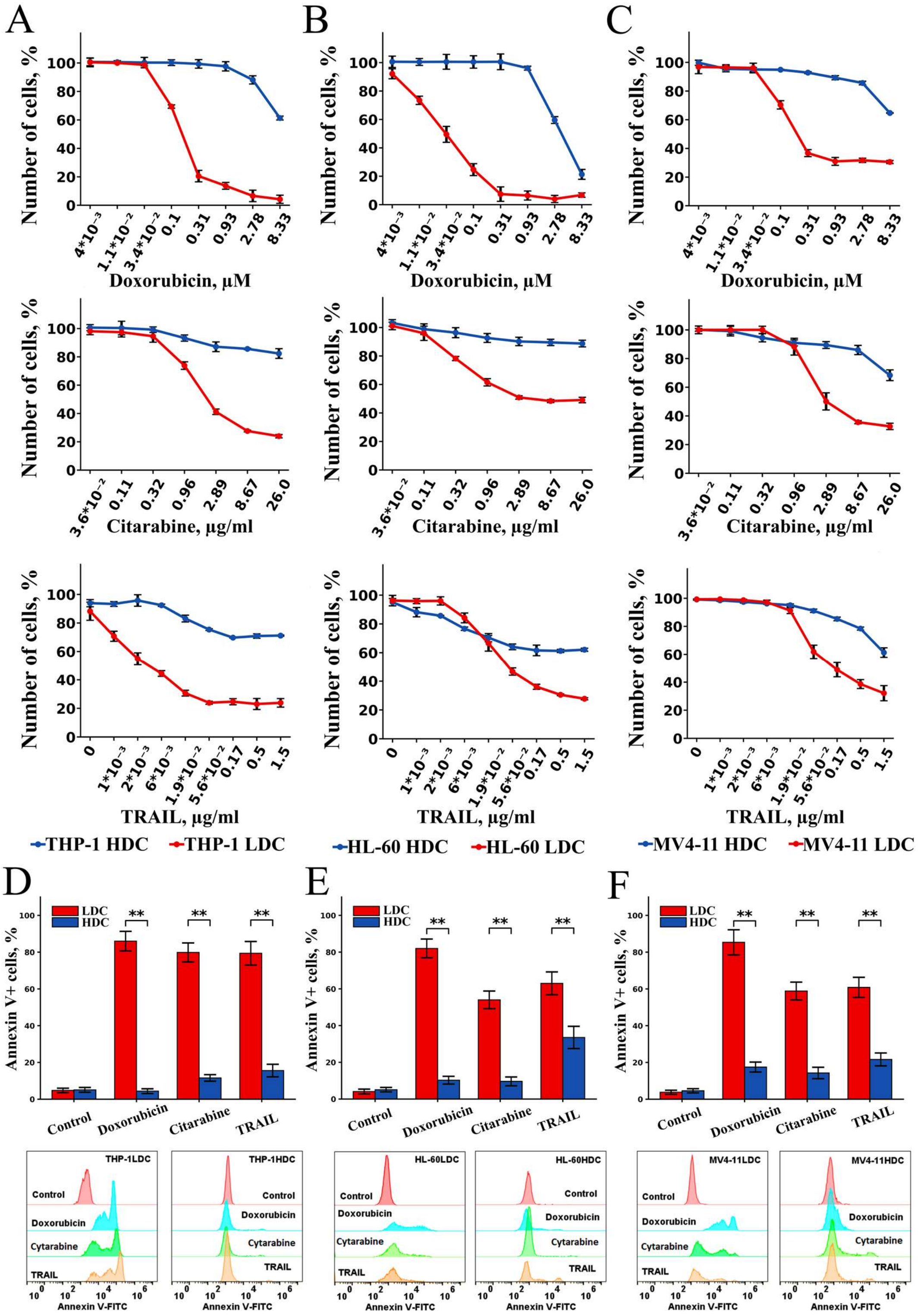

Figure S3. Cytotoxic effects of TRAIL, doxorubicin, and cytarabine on acute myeloid leukemia cells cultured at low (LDC) or high (HDC) density cultures. Viability of THP-1 (a), HL-60 (b), and MV4-11 (c) cells cultured at low (red) or high (blue) density after 24 hours of incubation with each drug. Proportion of Annexin V-positive THP-1 (d), HL-60 (e), and MV4-11 (f) cells grown at low (red) or high (blue) density after 24 hours of treatment. Data are presented as mean ± standard deviation ($n \geq 3$). * $p < 0.05$, compared to cell cultures in LDC.

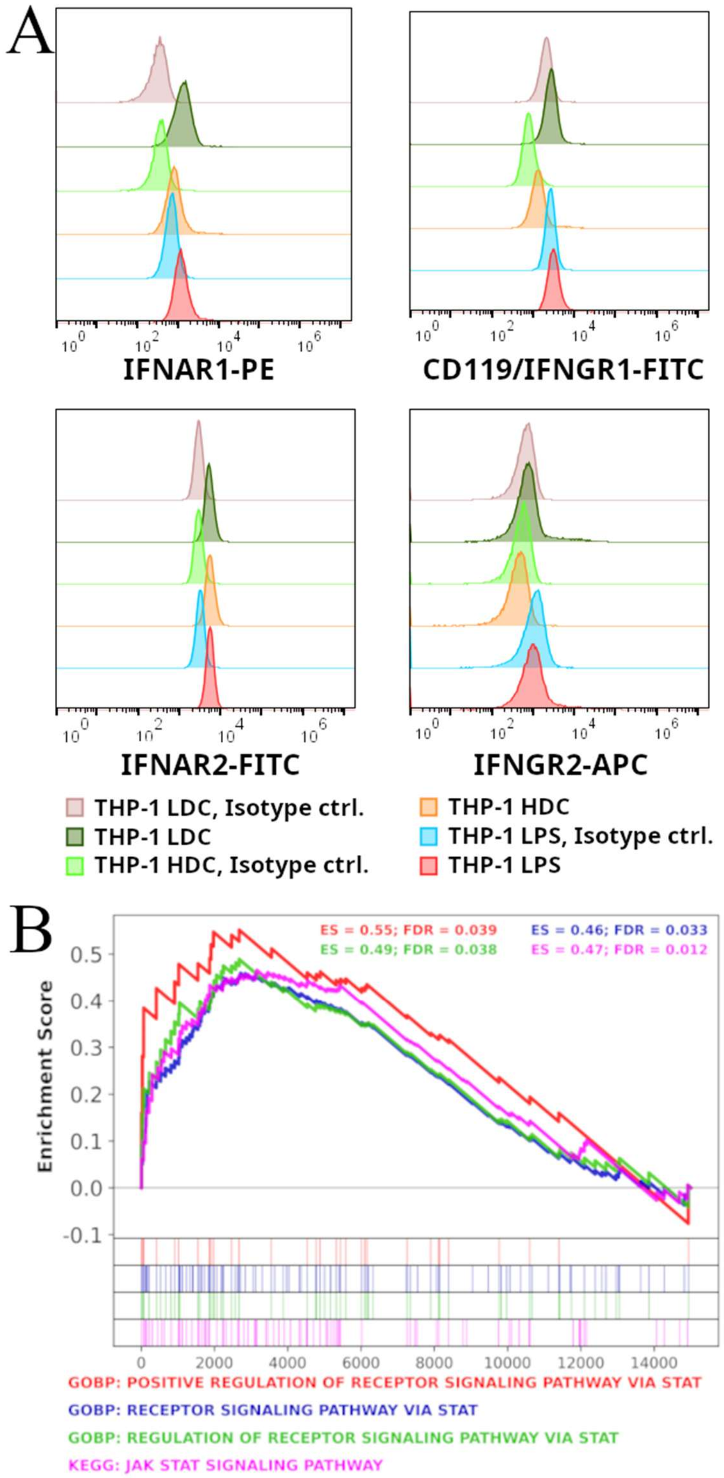

Figure S4. Interferon receptors surface expression and transcriptional JAK/STAT signaling pathway activation. A) Surface expression of receptors to interferon I (IFNAR1/2) (left panel) and interferon II (IFNGR1/2) (right panel) type. B) GSEA of gene sets of JAK/STAT signaling pathway from the Gene Ontology: Biological Process (GO:BP) and Kyoto Encyclopedia of Genes and Genomes (KEGG) collections in THP-1 HDC cells compared to THP-1 LDC cells. LDC – Low-Density Culture; HDC – High-Density Culture: LPS – Lipopolysaccharide.

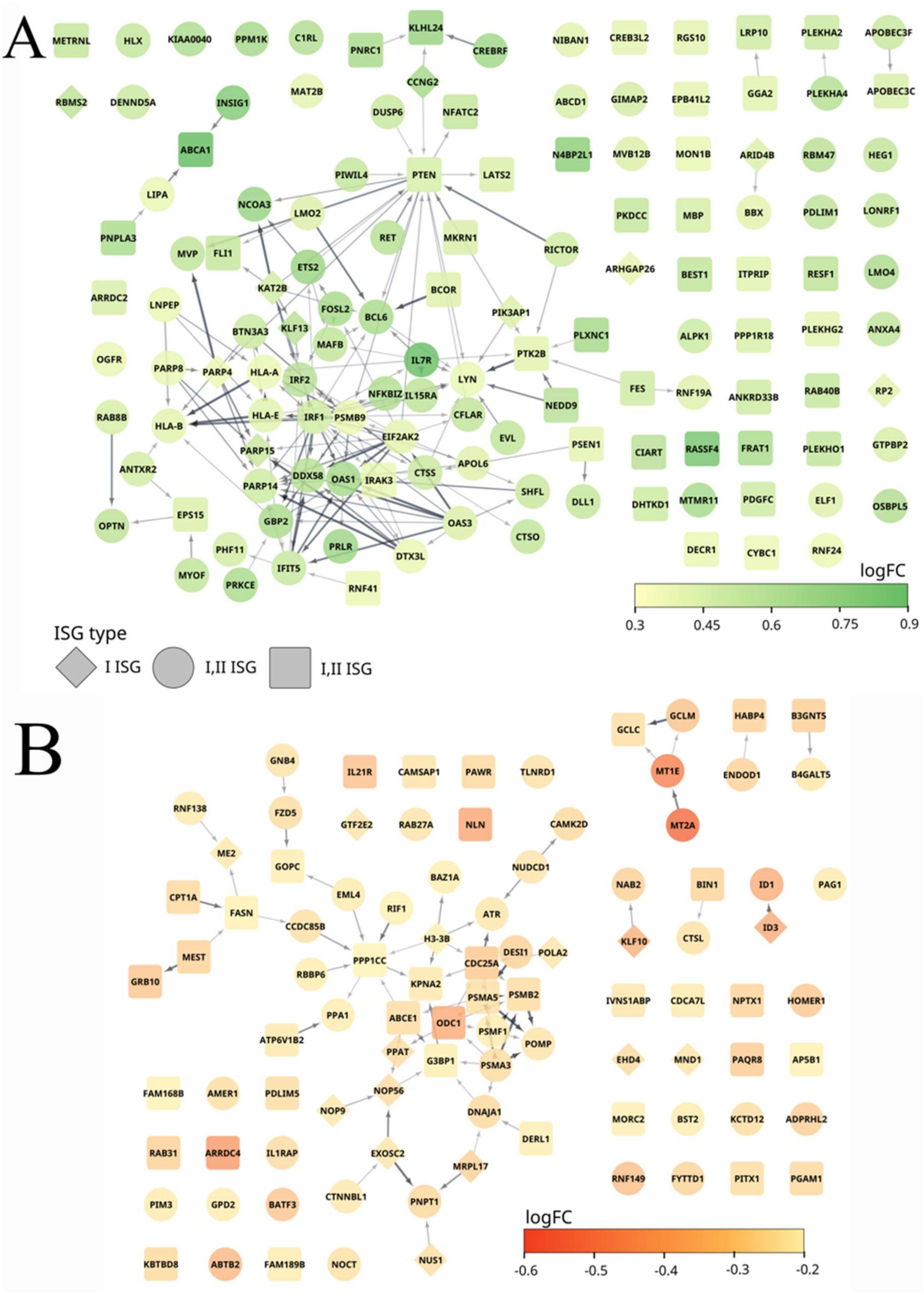

Figure S5. Full protein-protein interaction network of identified interferon-stimulated genes. PPI of ISG with UP (A) and DOWN (B) expression in THP-1 HDC compared to THP-1 LDC. FDR ≤ 0.05. LDC – Low-Density Culture; HDC – High-Density Culture.
